# Reduced-Dose vs Full-Dose Direct Oral Anticoagulants for Extended Anticoagulation Therapy in Patients With Venous Thromboembolism: A Systematic Review and Meta-Analysis of Randomized Controlled Trials

**DOI:** 10.1016/j.jscai.2026.105317

**Published:** 2026-04-09

**Authors:** Dhruvi K. Joshi, Najwaa Kirmani, Zaraith Coy, Siddharth P. Agrawal, Mir W. Majeed, Caroline Dagostin, Hritvik Jain, Zeeshan Mansuri, Peter A. Soukas, J. Dawn Abbott, Saraschandra Vallabhajosyula

**Affiliations:** a Department of Medicine, Narendra Modi Medical College and Sheth L.G. Hospital, Ahmedabad, India; b Deparment of Medicine, Dow Medical College, Karachi, Pakistan; c Department of Medicine, Universidad CES, Medellín, Colombia; d Department of Medicine, New York Medical College/Landmark Medical Center, Woonsocket, Rhode Island; e Department of Medicine, Government Medical College, Srinagar, India; f Department of Medicine, University of the Extreme South of Santa Catarina, Criciúma, Brazil; g Department of Medicine, All India Institute of Medical Sciences, Jodhpur, India; h Department of Medicine, G.C.S. Medical College, Ahmedabad, India; i Division of Cardiology, Department of Medicine, Warren Alpert Medical School of Brown University, Providence, Rhode Island; j Brown University Health Cardiovascular Institute, Providence, Rhode Island

**Keywords:** direct oral anticoagulants, extended anticoagulation, randomized controlled trials, reduced-dose, venous thromboembolism

## Abstract

**Background:**

Extended anticoagulation therapy is often required in patients with venous thromboembolism (VTE), but the optimal dosing strategy remains unclear. This study aimed to evaluate the efficacy and safety of reduced-dose versus full-dose direct oral anticoagulants in the extended treatment of VTE.

**Methods:**

We systematically searched PubMed, Embase, and Cochrane for randomized controlled trials (RCT) comparing reduced-dose versus full-dose direct oral anticoagulants for extended anticoagulation therapy in VTE. Primary outcomes included major bleeding, recurrent VTE, all-cause mortality, and clinically relevant nonmajor bleeding. Secondary outcomes included pulmonary embolism and upper limb deep vein thrombosis. Statistical analysis was performed using R 4.4.2. Quality assessment of included RCT was conducted using the Cochrane Risk of Bias 2 tool.

**Results:**

We included 8781 patients from 5 RCT with 50.1% (n = 4395) receiving reduced-dose anticoagulation; 21.7% (n = 1908) had active cancer, and 18.1% (n = 1585) had a history of previous deep vein thrombosis/pulmonary embolism. Major bleeding (1.2% vs 2.0%; risk ratio [RR], 0.62; 95% CI, 0.42-0.92; *P* = .019) and clinically relevant nonmajor bleeding (5.2% vs 7.0%; RR, 0.75; 95% CI, 0.63-0.88; *P* < .001) were significantly lower in the reduced-dose group. There was no significant difference in all-cause mortality (4.9% vs 5.8%; RR, 0.86; 95% CI, 0.63-1.17; *P* = .346) and recurrent VTE (2.0% vs 2.2%; RR, 0.92; 95% CI, 0.70-1.23; *P* = .587) between the 2 groups. All included studies were assessed as having an overall low risk of bias.

**Conclusions:**

Reduced-dose anticoagulation demonstrates significantly lower bleeding risk compared to full-dose in VTE patients, with comparable efficacy in preventing recurrent VTE and mortality.

## Introduction

Venous thromboembolism (VTE), which includes deep vein thrombosis (DVT) and pulmonary embolism (PE), is a major contributor to the global disease burden, causing significant disability and death.^1^ The anticoagulation dilemma in extended VTE treatment lies in balancing the risk-benefit profile of the optimal dose, drug choice, and treatment duration. Direct oral anticoagulants (DOAC) have become the preferred agents for both initial and extended anticoagulation because of their proven efficacy, favorable safety profile, and ease of use.^2^^,^^3^

Landmark trials such as AMPLIFY-EXT^2^ (Apixaban after the Initial Management of Pulmonary Embolism and Deep Vein Thrombosis with First-Line Therapy-Extended Treatment) and EINSTEIN CHOICE^3^ (Rivaroxaban or Aspirin for Extended Treatment of Venous Thromboembolism) demonstrated that reduced-dose DOAC effectively prevented VTE recurrence with favorable bleeding profiles, leading many clinicians to adopt dose deescalation strategies for extended anticoagulation. However, recent guidelines offer only a weak recommendation, based on very low certainty evidence, favoring reduced-dose apixaban or rivaroxaban over full-dose regimens in extended phase anticoagulation, underscoring the need for more robust synthesized evidence.^4^^,^^5^ Full-dose anticoagulants are commonly used, but they can increase bleeding risk, especially when taken for a long time. Recent studies have evaluated the efficacy and safety of reduced-dose versus full-dose DOAC for extended treatment of VTE.^6^, ^7^, ^8^ However, several knowledge gaps remain that limit the confident application of these findings across all patient populations requiring extended therapy. The recent publication of 2 large randomized controlled trials (RCT) specifically evaluating extended anticoagulation in cancer-associated thrombosis provides an opportunity for a more comprehensive evaluation of dose deescalation strategies.^6^^,^^8^ In this systematic review and meta-analysis of 5 RCT, we provide the most comprehensive evidence on the comparative outcomes of reduced-dose versus full-dose DOAC for extended VTE therapy, including subgroup analyses of clinically relevant patient populations.

## Methods

This systematic review and meta-analysis was conducted in accordance with the Cochrane Collaboration Handbook of Systematic Review of Interventions and the Preferred Reporting Items for the Systematic Reviews and Meta-analysis 2020 statement guidelines.^9^^,^^10^ The protocol was preregistered prospectively in PROSPERO (CRD420251045299) on May 4, 2025.

### Search strategy and inclusion criteria

We performed a systematic search of PubMed, Embase, and the Cochrane Central Register of Controlled Trials from January 2008 to May 2025, with the following search terms: “venous thromboembolism,” “VTE,” “Direct oral anticoagulants,” “Apixaban,” “Rivaroxaban,” “Betrixaban,” “Edoxaban,” “Reduced dose,” and “Dose reduction.” The complete search strategy is provided in Supplemental Table S1. Two authors (Z.C. and S.P.A.) independently screened titles and abstracts, followed by full-text assessment for eligibility. To identify additional studies, the reference lists of the included studies and previous meta-analyses and systematic reviews were manually reviewed to identify additional relevant trials. Any disagreements were resolved through discussion with a third author (D.K.J.).

We included all the studies that met the following inclusion criteria: (1) RCT, (2) enrolling patients with VTE who were given extended anticoagulation therapy with DOAC, (3) comparing reduced-dose DOAC versus full-dose DOAC, and (4) reporting at least 1 clinical outcome of interest. We excluded the following studies: (1) published as editorials, letters, or conference abstracts, (2) with no control group, (3) with observational or cohort study design, and (4) with overlapping populations.

### Data extraction and outcomes

Two authors (D.K.J. and Z.C.) independently extracted data, using predefined search criteria and quality assessment. Extracted variables included study characteristics (author and year of publication), population characteristics (sample size, mean age, sex distribution, history of previous DVT or PE, active cancer status, and proportion of unprovoked index VTE), intervention arm details, comparison arm details, and all prespecified outcomes of interest. The risk of bias and quality assessment was evaluated using version 2 of the Cochrane Risk of Bias assessment tool (RoB 2).^11^ Two authors (N.K. and H.J.) independently completed the risk of bias assessment. Discrepancies were resolved by consensus with a third author.

Primary outcomes included major bleeding, recurrent VTE, all-cause mortality, and clinically relevant nonmajor bleeding. Secondary outcomes included composite bleeding, PE, and upper limb DVT. Subgroup analyses were performed for recurrent VTE and the composite of major bleeding and clinically relevant nonmajor bleeding by age, sex, body mass index, renal function, active cancer status, DVT status, and baseline anticoagulation drug type to assess potential effect modification across these clinically relevant patient characteristics.

### Statistical analysis

All statistical analyses were performed using R version 4.4.2 (R Foundation for Statistical Computing). We used risk ratio (RR) with 95% CI as the measure of effect size to compare binary outcomes. *P* < .05 was considered statistically significant. Heterogeneity was assessed with the Cochran Q test and quantified with the I^2^ statistic, with values of <25%, 25% to 50%, 50% to 75%, and >75% representing low, moderate, high, and very high heterogeneity, respectively.^9^ Wherever notable heterogeneity was observed, we performed a leave-one-out sensitivity analysis by sequentially excluding each study to assess its influence on the overall effect estimates and ensure the robustness of our findings. We used the restricted maximum likelihood random-effects model. RR were used as the primary effect measure for the meta-analysis, as they were consistently reported across all included studies. Wherever available, hazard ratios (HR) were extracted and included in a sensitivity analysis presented in the Supplement. To explore potential sources of heterogeneity and effect modification, we performed meta-regression analyses using median follow-up duration as the variable for all primary and secondary outcomes.

## Results

### Study selection and baseline characteristics

**Figure 1:**
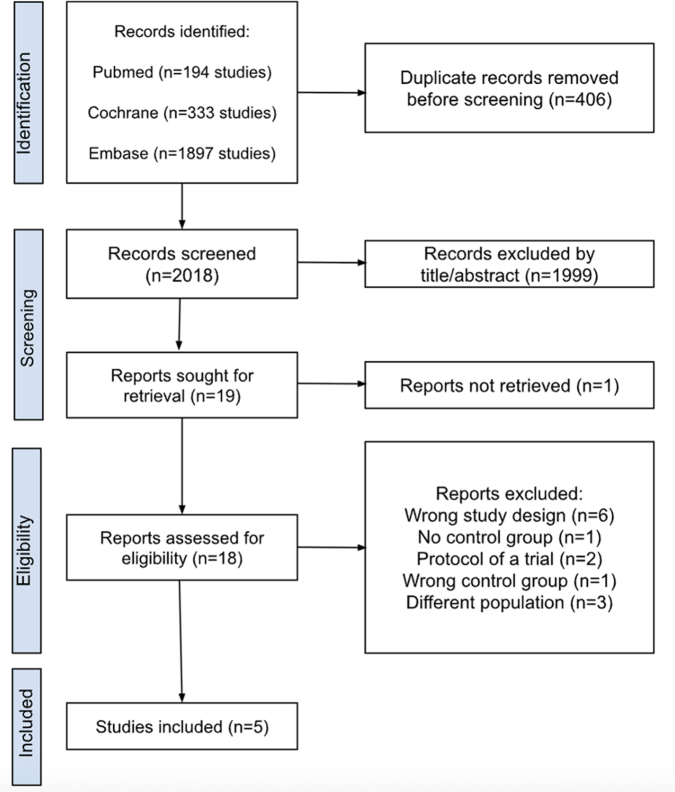
**Preferred Reporting Items for Systematic reviews and Meta-Analyses (PRISMA) flowchart of study screening and selection.**

The initial search yielded 2424 results. After removal of duplicate records and ineligible studies, 18 remained and were fully reviewed based on the inclusion criteria. Of these, a total of 5 RCT were included, comprising 8781 patients (Figure 1).^2^^,^^3^^,^^6^, ^7^, ^8^ A total of 50.1% (n = 4395) of patients received reduced-dose anticoagulation, whereas 49.9% (n = 4386) received full-dose anticoagulation. Study characteristics are reported in Table 1. The proportion of female patients was 44.2% (n = 3878), while 21.7% (n = 1908) had active cancer and 18.1% (n = 1585) had a history of previous DVT/PE (Table 1). Risk of bias was assessed using RoB 2.^11^ All included studies were judged to have an overall low risk of bias (Supplemental Figure S1).

**Table 1:** Baseline characteristics of all included studies

Study, year	Location	Reduced-dose	Full-dose	Reduced/full								
				Total, N	Age, y	Female patients, N	BMI, kg/m^2^	Previous DVT/PE, N	Active cancer, N	Unprovoked index VTE, N	Known thrombophilia, N	Median follow-up period, mo
Agnelli et al,^2^ 2013	Multicenter (28 countries)	Apixaban: 2.5 mg	Apixaban: 5 mg	840/813	56.6 ± 15.3/56.4 ± 15.6	353/344	29.3 ± 6.1/29.2 ± 5.7	99/118	15/9	783/737	–	12
Weitz et al,^3^ 2017	Multicenter (31 countries)	Rivaroxaban: 10 mg	Rivaroxaban: 20 mg	1127/1107	58.8 ± 14.7/57.9 ± 14.7	507/505	BMI <30: 712/751; BMI >30: 394/376	197/198	27/25	480/441	74/79	12
Couturaud et al,^6^ 2025	France	Apixaban: 2.5 mg Rivaroxaban: 10 mg	Apixaban: 5 mg Rivaroxaban: 20 mg	1383/1385	62.2 ± 14.3/63.1 ± 14.3	489/481	28.6 ± 7.6/28.3 ± 5.1	316/296	34/37	836/845	234/234	37.1
McBane et al,^7^ 2024	USA	Apixaban: 2.5 mg	Apixaban: 5 mg	179/181	63.6 ± 11.0/64.3 ± 10.7	92/107	29.2 ± 6.6/29.5 ± 6.8	18/16	–	–	–	12
Mahé et al,^8^ 2025	Multicenter (11 countries)	Apixaban: 2.5 mg	Apixaban: 5 mg	866/900	67.2 ± 11.0/67.7 ± 11.4	491/509	27.0 ± 5.3/27.0 ± 5.4	157/170	864/897	–	–	12

Values are mean ± SD unless otherwise stated.

DVT, deep venous thrombosis; FB, fatal bleeding; PE, pulmonary embolism; VTE, venous thromboembolism.

### Bleeding outcomes

**Figure 2:**
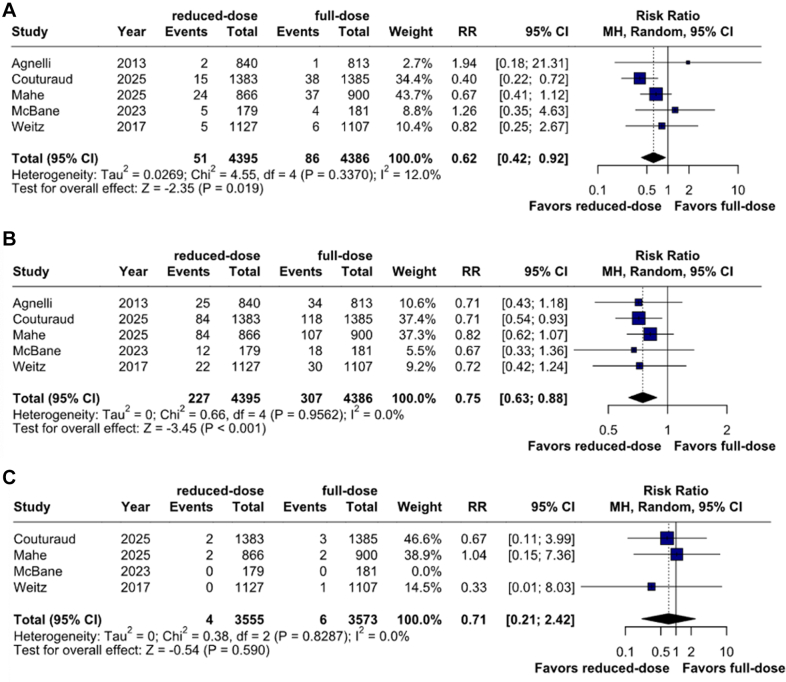
**Bleeding outcomes.** (**A**) Major bleeding risk was significantly reduced in reduced-dose compared to full-dose direct oral anticoagulants; (**B**) clinically relevant nonmajor bleeding risk was significantly reduced in reduced-dose compared to full-dose direct oral anticoagulants; (**C**) fatal bleeding risk was not significant between both groups. MH, Mantel-Haenszel; RR: risk ratio.

Reduced-dose anticoagulation was associated with a statistically significant reduction in the risk of major bleeding (1.2% vs 2.0%; RR, 0.62; 95% CI, 0.42-0.92; *P* = .019; I^2^ = 12%; Figure 2A) and clinically relevant nonmajor bleeding (5.2% vs 7.0%; RR, 0.75; 95% CI, 0.63-0.88; *P* < .001; I^2^ = 0%; Figure 2B) compared to full-dose anticoagulation. Rates of fatal bleeding (0.1% vs 0.2%; RR, 0.71; 95% CI, 0.21-2.42; *P* = .590; I^2^ = 0%; Figure 2C) did not differ significantly between patients receiving reduced-dose versus full-dose anticoagulation.

### Efficacy outcomes

**Figure 3:**
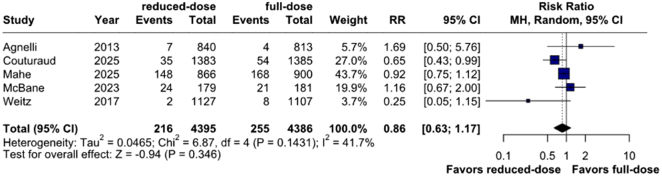
**Mortality outcomes between reduced-dose and full-dose direct oral anticoagulants.** MH, Mantel-Haenszel; RR, risk ratio.

**Figure 4:**
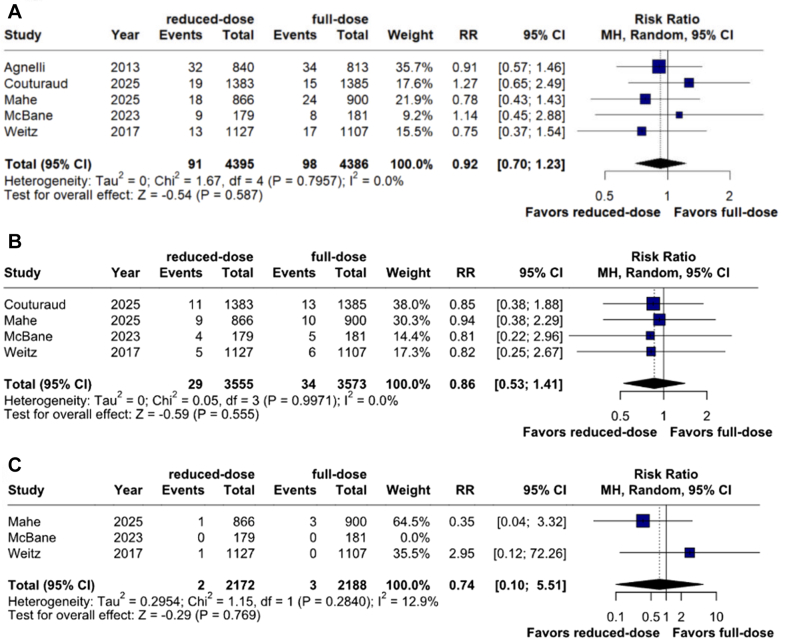
**Efficacy outcomes.** (**A**) Recurrent venous thromboembolism risk was comparable between both groups; (**B**) pulmonary embolism risk was not significant between both groups; (**C**) upper limb deep vein thrombosis risk was not significant between both groups. MH, Mantel-Haenszel; RR, risk ratio.

The risk of all-cause mortality (4.9% vs 5.8%; RR, 0.86; 95% CI, 0.63-1.17; *P* = .346; I^2^ = 42%; Figure 3) and recurrent VTE (2.0% vs 2.2%; RR, 0.92; 95% CI, 0.70-1.23; I^2^ = 0%; *P* = .587; Figure 4A) were not significantly different between groups. There was no significant difference in risks of PE (0.8% vs 1.0%; RR, 0.86; 95% CI, 0.53-1.41; *P* = .555; I^2^ = 0%; Figure 4B) and upper limb DVT (0.1% vs 0.1%; RR, 0.74; 95% CI, 0.10-5.51; *P* = .769; I^2^ = 13%; Figure 4C) between the 2 cohorts. Pooled analyses of outcomes reported as HR are detailed in Supplemental Figure S2.

### Subgroup analyses

We performed subgroup analyses for recurrent VTE and the composite of major bleeding and clinically relevant nonmajor bleeding according to key study-level characteristics. We examined recurrent VTE across subgroups defined by renal function (creatinine clearance: <50, 50-79, and ≥80 mL/min), age, baseline anticoagulation drug type, sex, body mass index (<30, ≥30 kg/m^2^), and DVT status.

For recurrent VTE, in subgroup analyses stratified by sex, there was significant effect modification (*P*_interaction_ = .014). The ratio of odds ratios (female vs male) was 2.22 (95% CI, 1.17-4.20), indicating that the treatment effect differed significantly by sex, with reduced-dose anticoagulation showing benefit in male patients but not in female patients. No statistically significant effect modification was seen across the remaining prespecified subgroups for this outcome (Supplemental Figures S3-S9). The Central Illustration summarizes the subgroup analysis for recurrent VTE.

**Central Illustration:**
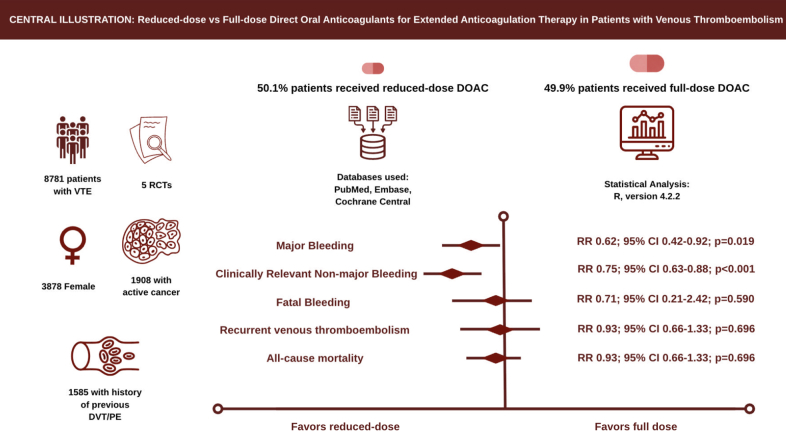
**Reduced-dose versus full-dose direct oral anticoagulants for extended anticoagulation therapy in patients with venous thromboembolism.** DOAC, direct oral anticoagulants; DVT, deep vein thrombosis; PE, pulmonary embolism; RCT, randomized controlled trials; RR, relative risk; VTE, venous thromboembolism.

We examined the composite of major bleeding and clinically relevant nonmajor bleeding across subgroups defined by renal function (creatinine clearance: <50, 50-79, and ≥80 mL/min), age, baseline anticoagulation drug type, sex, active cancer, and DVT status. In subgroup analyses stratified by sex, reduced-dose anticoagulation was associated with a lower risk of the composite of major and clinically relevant nonmajor bleeding in both women and men, with no statistically significant between-subgroup difference across both groups. In subgroup analyses stratified by creatinine clearance, reduced-dose anticoagulation significantly reduced the composite of major and clinically relevant nonmajor bleeding in patients with creatinine clearance 50 to 79 and ≥80 mL/min, whereas no significant difference was observed in those with creatinine clearance <50 mL/min. In analyses stratified by active cancer status, reduced-dose anticoagulation significantly reduced the composite of major and clinically relevant nonmajor bleeding in both patients with and without active cancer, with the effect being consistent across subgroups. In analyses stratified by anticoagulant type, reduced-dose therapy significantly reduced the composite of major and clinically relevant nonmajor bleeding with both apixaban and rivaroxaban, and the effect was consistent across the subgroups. No statistically significant interaction was detected across creatinine clearance categories, active cancer status, or anticoagulant type, suggesting broad consistency of treatment effect across these clinically relevant subgroups. Although the overall effect was statistically significant, showing reduced risk of composite bleeding outcome with reduced-dose anticoagulation, upon performing stratified subgroup analysis, these findings in patients with PE with or without DVT became nonsignificant, whereas in patients with isolated proximal DVT continued to remain statistically significant. The effect of reduced-dose versus full-dose anticoagulation was consistent across all other subgroups (Supplemental Figures S10-S15). Supplemental Figures S16 and S17 summarize the subgroup analysis for recurrent VTE and composite bleeding outcome, respectively. The Central Illustration summarizes the key findings of this study.

### Sensitivity analyses

To assess the robustness of the major bleeding outcome, we performed a leave-one-out sensitivity analysis despite low heterogeneity, iteratively removing 1 study at a time to ensure that the findings were not driven by any single trial. Overall, the pooled estimate for major bleeding remained consistent with the exclusion of each individual study. Leave-one-out sensitivity analysis was also performed for all-cause mortality because of high heterogeneity; exclusion of individual studies did not significantly affect the overall estimate. Results are included in the Supplemental Figure S18.

### Meta-regression analyses

Meta-regression analyses evaluating follow-up duration as a study-level covariate demonstrated no statistically significant modification of the treatment effect for any clinical outcome (Table 2). For clinically relevant nonmajor bleeding, fatal bleeding, the composite bleeding end point, PE, and recurrent VTE, the regression coefficients were small and nonsignificant (*P* > .40), with R^2^ values of 0%, indicating that follow-up duration explained none of the between-study heterogeneity. For major bleeding (analyzed using both RR and HR), follow-up duration showed a borderline inverse association (β ≈ –0.03, *P* = .069), suggesting a potential trend toward greater bleeding reduction with reduced-dose DOAC in longer-duration studies; however, these findings did not reach statistical significance and should be interpreted cautiously. Overall, these results indicate that the comparative efficacy and safety of reduced- versus full-dose DOAC during extended therapy appear consistent across the range of follow-up durations observed in the included trials (Supplemental Figures S19-S26).

**Table 2:** Meta-regression with follow-up duration as a study-level covariate

Outcome (effect size)	Covariate	Regression coefficient (β)	*P* value	R^2^	Interpretation	Supplemental figure
CRNMB (RR)	Follow-up	–0.003	.68	0	No influence of follow-up duration on CRNMB. The effect was consistent across studies	S21
Fatal bleeding (RR)	Follow-up	–0.037	.53	0	No association between follow-up duration and FB risk. Treatment effects remained stable over time.	S22
Major bleeding (RR)	Follow-up	–0.032	.069	100	A borderline trend suggested lower major bleeding with longer follow-up, but this was not statistically significant.	S23
Composite of major bleeding and CRNMB (RR)	Follow-up	–0.018	.41	0	Follow-up duration had no effect on the composite bleeding outcome. Results were consistent across studies.	S24
Major bleeding (HR)	Follow-up	–0.032	.069	100	A borderline trend suggested lower major bleeding (HR) with longer follow-up, but this was not statistically significant.	S20
Mortality (RR)	Follow-up	–0.018	.11	99.996	Longer follow-up showed a nonsignificant tendency toward lower mortality; no confirmed effect modification.	S25
PE (RR)	Follow-up	–0.032	.62	0	No relationship between follow-up duration and PE risk. Effects were stable across study durations.	S26
Recurrent VTE (RR)	Follow-up	–0.004	.95	0	Follow-up duration had no impact on recurrent VTE risk. There was no evidence of effect modification.	S19

CRNMB, clinically relevant nonmajor bleeding; HR, hazard ratio; PE, pulmonary embolism; RR, risk ratio; VTE, venous thromboembolism.

## Discussion

In this systematic review and meta-analysis of 5 RCT with 8781 patients, we compared reduced-dose versus full-dose anticoagulation extended therapy in patients with VTE. The main findings with reduced-dose include: (1) significant reduction in major bleeding and clinically relevant nonmajor bleeding; (2) mortality and incidence of recurrent VTE did not differ significantly between both cohorts; (3) fatal bleeding, PE, and upper limb DVT risk reduction did not differ significantly compared to the full-dose cohort (Central Illustration).

Current clinical guidelines, including the 2020 American Society of Hematology recommendations, endorse extended phase anticoagulation for patients with unprovoked or recurrent VTE, particularly when the risk of recurrence outweighs the risk of bleeding. However, optimal dosing of DOAC for extended thromboprophylaxis in patients with VTE remains a subject of ongoing debate, largely because of the lack of evidence.^2^^,^^3^^,^^12^ Our meta-analysis demonstrated a significant reduction in major bleeding risk (1.2% vs 2.0%) with dose reduction compared to full-dose anticoagulation. These findings are important because bleeding is a dreaded complication of being on anticoagulation therapy and can lead to treatment discontinuation, hospitalization, and increased mortality. A prior meta-analysis^13^ analyzed bleeding events (n = 5842) which showed that reduced-dose did not show a difference in major or clinically relevant nonmajor bleeding from full-dose anticoagulation (*P* = .09). As the sample size increased in our meta-analysis, the benefit-risk profile increasingly favored reduced-dose anticoagulation, suggesting that larger scale future studies may help establish the true clinical impact of dose deescalation.

In our subgroup analyses based on creatinine clearance, the reduced bleeding risk with reduced-dose DOAC was seen only in patients with creatinine clearance 50 to 79 and ≥80 mL/min, but not in those with creatinine clearance <50 mL/min. This is biologically plausible. In severe renal impairment, baseline bleeding risk is already high, and DOAC tend to accumulate regardless of the nominal dose.^14^ In contrast, among patients with preserved or mildly impaired renal function (creatinine clearance ≥50 mL/min), drug clearance is more predictable, and the separation in drug exposure between full- and reduced-dose regimens is clearer. In this setting, lowering the dose is more likely to translate into a meaningful reduction in bleeding without a large loss of efficacy, which is consistent with our findings.

Notably, we observed significant effect modification by sex for recurrent VTE (*P*_interaction_ = .014). Reduced-dose anticoagulation was associated with lower VTE recurrence in male patients (OR, 0.64; 95% CI, 0.43-0.97) but not in female patients (OR, 1.42; 95% CI, 0.87-2.32). This finding warrants cautious interpretation given that randomization in the included trials was not stratified by sex, and the observed interaction could reflect chance imbalances in baseline characteristics. Although sex-based differences in pharmacokinetics are recognized, the mechanisms underlying differential treatment responses between sexes remain incompletely understood.

Extended reduced-dose anticoagulation may lower bleeding risk without compromising efficacy, as shown in this meta-analysis. In secondary prevention of VTE, optimizing anticoagulation dosing is critical to reduce morbidity, mortality, and prevent disease progression.^15^ Identifying patients at high bleeding risk and applying mitigation strategies are essential for both short- and long-term VTE management.^12^ Lower bleeding risk observed with reduced-dose anticoagulation may extend across diverse patient subgroups, as 21.7% (n = 1908) had active cancer and 18.1% (n = 1585) had a history of prior DVT/PE in this meta-analysis. However, dedicated subgroup analyses are needed to draw more definitive conclusions. This meta-analysis found that reduced-dose DOAC were comparable to full-dose regimens in preventing recurrent VTE. These findings are further supported by recent real-world evidence from a large observational registry,^16^ which demonstrated that dose reduction preserves low recurrence rates while reducing bleeding risk, an effect most pronounced among patients with cancer. There was inclusion of a substantial cancer population (18.1%, n = 1585) in this meta-analysis, predominantly from the Mahé et al^8^ and EVE trial.^7^ The cancer cohorts in these trials were heterogeneous, including patients with solid tumors across gastrointestinal, lung, genitourinary, and breast malignancies. Importantly, a subset had experienced prior VTE events, representing a particularly high-risk population for recurrence. In the Mahé et al^8^ trial (n = 1766), the most common cancer types were breast (22.7%), gastrointestinal (15.3%), and lung (11.2%) malignancies. A majority (99.7%) had active cancer, and 15% had a history of prior VTE (18.5%). In the EVE trial^7^ (n = 360), gastrointestinal (24.7%), leukemia/lymphoma/myeloma (13.8%), and lung (11.9%) cancers were most common, with 9.4% patients having a history of prior VTE. In subgroup analyses stratified by active cancer, reduced-dose anticoagulation was associated with a lower risk of the composite of major bleeding and clinically relevant nonmajor bleeding in both cancer and noncancer patients. For recurrent VTE, there was no significant difference between reduced- and full-dose therapy in either cancer subgroup. However, given the heterogeneity in cancer types, stages, and concurrent therapies, dedicated analyses examining outcomes across cancer-specific subgroups would be valuable to guide individualized treatment decisions in this complex population.

Our findings build on prior meta-analyses.^17^^,^^18^ We conducted detailed subgroup analyses and meta-regression—components that were not reported in earlier meta-analyses, providing a more robust assessment of potential effect modifiers and strengthening the methodological rigor of our study. However, this meta-analysis has important limitations. Firstly, although only 5 RCT were included, the overall sample size was substantial (n = 8781), which helped to enhance the robustness of the findings. Secondly, subgroup analyses for specific patient populations such as older individuals, those with renal impairment, high bleeding risk, or cancer-associated thrombosis, were not conducted because of the lack of consistently reported data across these categories. Thirdly, because of the small number of eligible RCT (n = 5), statistical methods for publication bias, such as funnel plots or Egger’s test, could not be performed. To reduce the risk of missing studies, we conducted a comprehensive and systematic literature search across multiple databases and checked the reference lists of relevant reviews. Meta-regression analyses were limited by the small number of included studies (n = 5). Finally, there was heterogeneity in the specific DOAC included across the studies, which limited our ability to perform direct comparisons between individual agents within the class.

## Conclusions

As the landscape of VTE management continues to evolve, our results suggest that reduced-dose DOAC may provide a more favorable safety profile while maintaining comparable effectiveness in preventing the recurrence of thromboembolic events, supporting the growing consideration of dose deescalation as a viable strategy for extended anticoagulation in selected patients. Future studies should aim to define which patient subgroups benefit most from reduced-dose anticoagulation and help guide individualized treatment decisions in extended VTE therapy.

## Declaration of competing interest

J. Dawn Abbott receives research funding from Boston Scientific, Shockwave Medical, MedAlliance, and serves as a consultant for Abbott, Medtronic, Penumbra, and Recor Medical. All other authors reported no financial interests.

## Footnotes

To access the supplemental material accompanying this article, visit the online version of the *Journal of the Society for Cardiovascular Angiography & Interventions* at 10.1016/j.jscai.2026.105317.
