# Supplementary material for: Reduced-Dose vs Full-Dose Direct Oral Anticoagulants for Extended Anticoagulation Therapy in Patients With Venous Thromboembolism: A Systematic Review and Meta-Analysis of Randomized Controlled Trials
Source: J Soc Cardiovasc Angiogr Interv. 2026 Apr 9;5(5):105317. doi: 10.1016/j.jscai.2026.105317 (PMC13198125; doi:10.1016/j.jscai.2026.105317)
Supplement: Supplementary Material [file mmc1.docx]

**SUPPLEMENTARY MATERIAL**

**Reduced-dose vs Full-dose Direct Oral Anticoagulants for Extended Anticoagulation Therapy in Patients with Venous Thromboembolism: A Systematic Review and Meta–Analysis of Randomized Controlled Trials**

Dhruvi K Joshi, MBBS; Najwaa Kirmani, MBBS; Zaraith Coy, MD; Siddharth P Agrawal, MD; Mir W Majeed, MBBS; Caroline Dagostin, MD, MSc; Hritvik Jain, MBBS; Zeeshan Mansuri, MD; Peter A Soukas, MD; J Dawn Abbott, MD; Saraschandra Vallabhajosyula, MD MSc

**SUPPLEMENTARY TABLES**

**Supplementary Table S1: Detailed search strategy for PubMed, Cochrane Central and Embase databases**

| **PUBMED**  ("Venous Thromboembolism"[Mesh] OR VTE OR "Deep vein thrombosis" OR "Venous Thrombosis"[Mesh] OR "Pulmonary Embolism"[Mesh] OR PE OR "Venous thromboembolic disease" OR DVT OR thromboembolism) AND ("Factor Xa Inhibitors"[Mesh] OR "Antithrombins"[Mesh] OR "direct oral anticoagulants" OR DOAC OR "Non-Vitamin K Antagonist Oral Anticoagulants" OR apixaban OR rivaroxaban OR dabigatran OR edoxaban OR betrixaban) AND ("reduced dose" OR "reduced-dose" OR "Low dose" OR "lower dose" OR "tapered dose" OR "smaller dose" OR "lesser dose" OR "decreased dose" OR "dose reduction" OR "titrated dose" OR "dose titration" OR "dose adjustment" OR "fractional dose" OR "De-escalated dose" OR "dose de-escalation" OR "dose reduction" OR "Modified dosing regimen") AND (randomized controlled trial[pt] OR controlled clinical trial[pt] OR clinical trials as topic[mesh:noexp] OR trial[ti] OR random*[tiab] OR placebo*[tiab]) |
| --- |
| **COCHRANE CENTRAL**  ("Venous Thromboembolism" OR VTE OR "Deep vein thrombosis" OR "Venous Thrombosis" OR "Pulmonary Embolism" OR PE OR "Venous thromboembolic disease" OR DVT OR thromboembolism) AND ("Factor Xa Inhibitors" OR "Antithrombins" OR "direct oral anticoagulants" OR DOAC OR "Non-Vitamin K Antagonist Oral Anticoagulants" OR apixaban OR rivaroxaban OR dabigatran OR edoxaban OR betrixaban) AND ("reduced dose" OR "reduced-dose" OR "Low dose" OR "lower dose" OR "tapered dose" OR "smaller dose" OR "lesser dose" OR "decreased dose" OR "dose reduction" OR "titrated dose" OR "dose titration" OR "dose adjustment" OR "fractional dose" OR "De-escalated dose" OR "dose de-escalation" OR "dose reduction" OR "Modified dosing regimen")] |
| **EMBASE**  ('venous thromboembolism'/exp OR 'venous thromboembolism' OR vte OR 'venous thrombosis'/exp OR 'venous thrombosis' OR 'thromboembolism'/exp OR thromboembolism OR 'deep vein thrombosis'/exp OR 'deep vein thrombosis' OR dvt OR 'pulmonary embolism'/exp OR 'pulmonary embolism' OR 'pe'/exp OR pe OR 'venous thromboembolic disease') AND ('factor xa inhibitors'/exp OR 'factor xa inhibitors' OR 'direct factor xa inhibitors'/exp OR 'direct factor xa inhibitors' OR 'direct thrombin inhibitors' OR 'direct oral anticoagulants' OR doac OR 'novel oral anticoagulants' OR noac OR 'non-vitamin k antagonist oral anticoagulants' OR 'apixaban'/exp OR apixaban OR 'rivaroxaban'/exp OR rivaroxaban OR 'dabigatran'/exp OR dabigatran OR 'edoxaban'/exp OR edoxaban OR 'betrixaban'/exp OR betrixaban) AND ('reduced dose' OR 'reduced-dose' OR 'low dose'/exp OR 'low dose' OR 'lower dose' OR 'tapered dose' OR 'smaller dose' OR 'lesser dose' OR 'decreased dose'  OR 'titrated dose' OR 'dose titration'/exp OR 'dose titration' OR 'dose adjustment'/exp OR 'dose adjustment' OR 'fractional dose'/exp OR 'fractional dose' OR 'de-escalated dose' OR 'dose de-escalation' OR 'dose reduction'/exp OR 'dose reduction' OR 'modified dosing regimen') |

**SUPPLEMENTARY FIGURES**

**Supplementary Figure S1: Risk of bias assessment using RoB-2 tool**


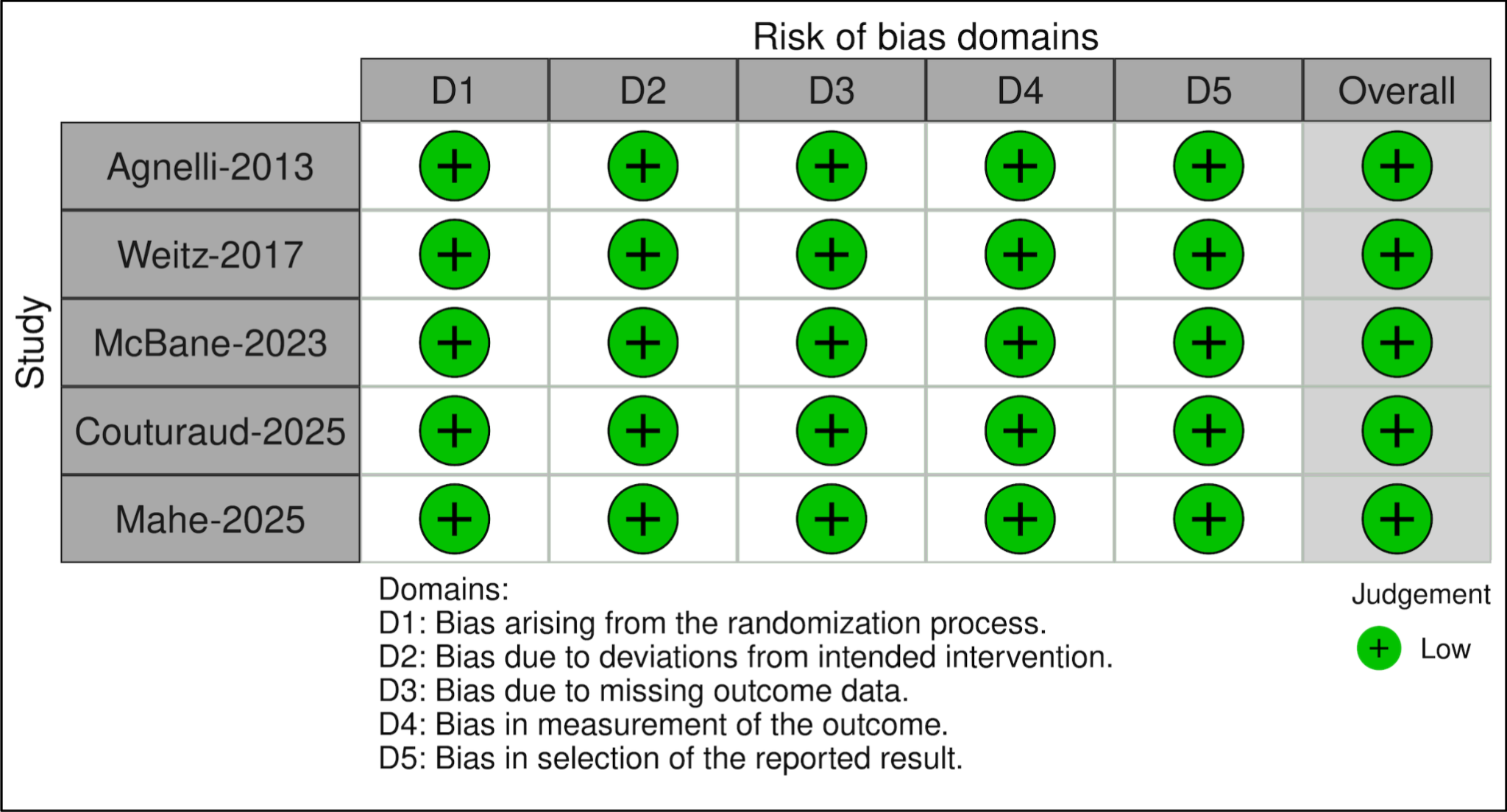


**Supplementary Figure S2: Forest plots for pooled hazard ratios for key clinical outcomes**


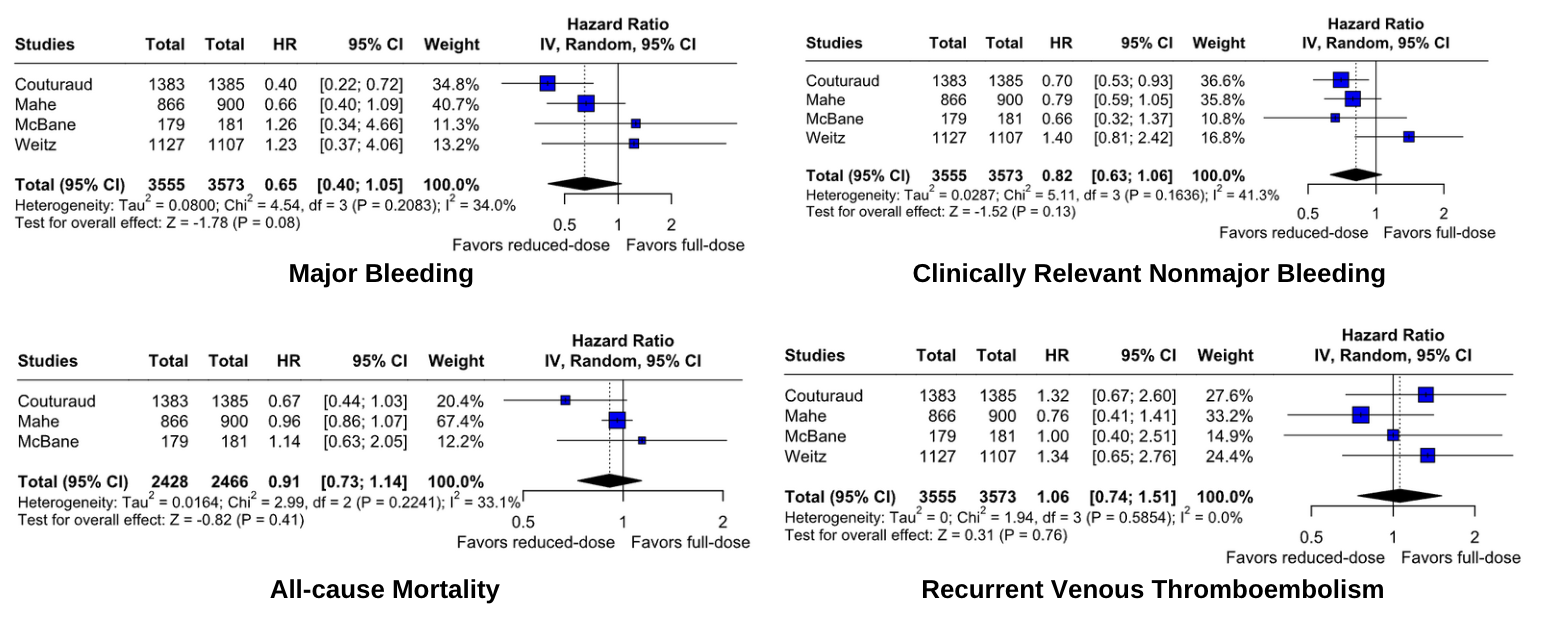


**Supplementary Figure S3: Subgroup analyses for recurrent Venous Thromboembolism by Sex**


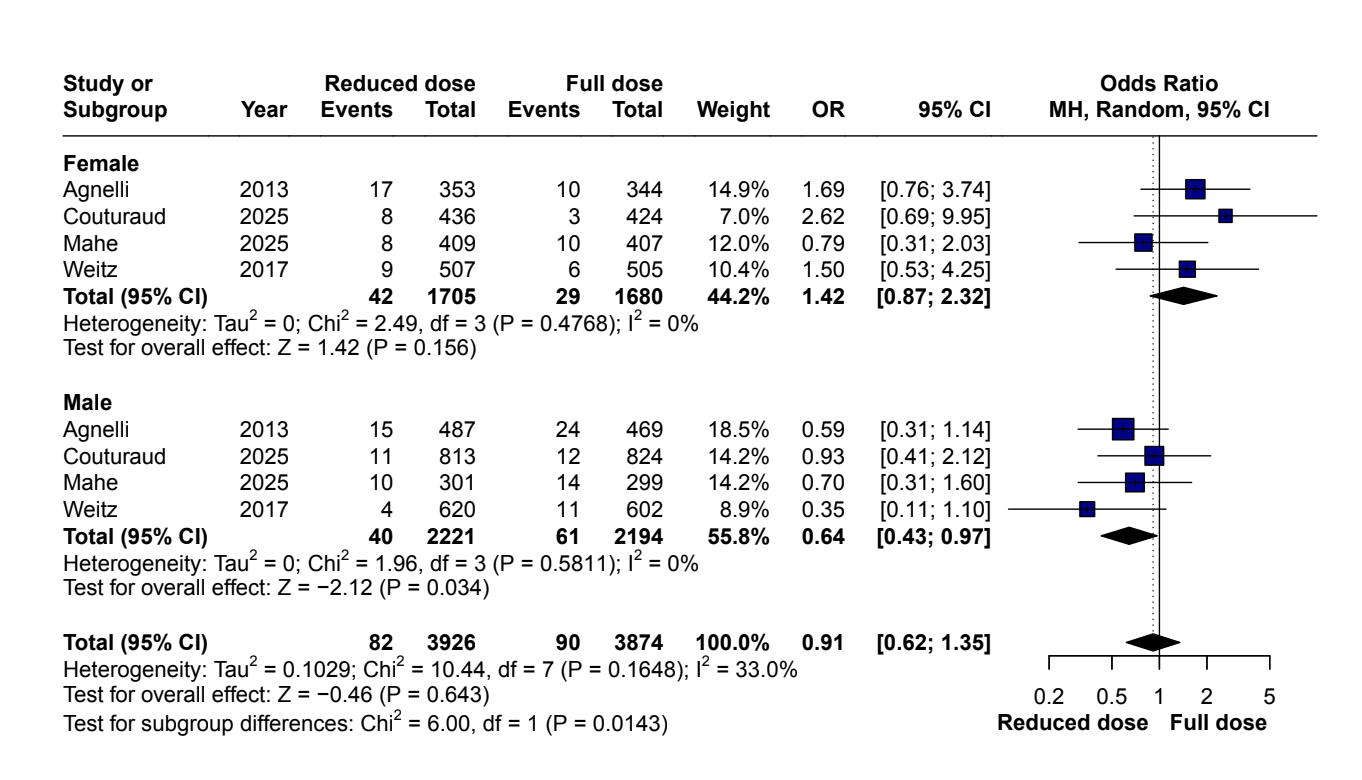


Figure S3: Subgroup analyses for recurrent venous thromboembolism by sex. P-interaction = 0.014, indicating significant effect modification by sex. Ratio of odds ratios (female vs male) = 2.22 (95% CI 1.17-4.20). Absolute risk difference was +0.74% (95% CI −0.2% to +1.7%) in females and −0.98% (95% CI −1.9% to −0.1%) in males.

**Supplementary Figure S4: Subgroup analyses for recurrent Venous Thromboembolism by Age**

**
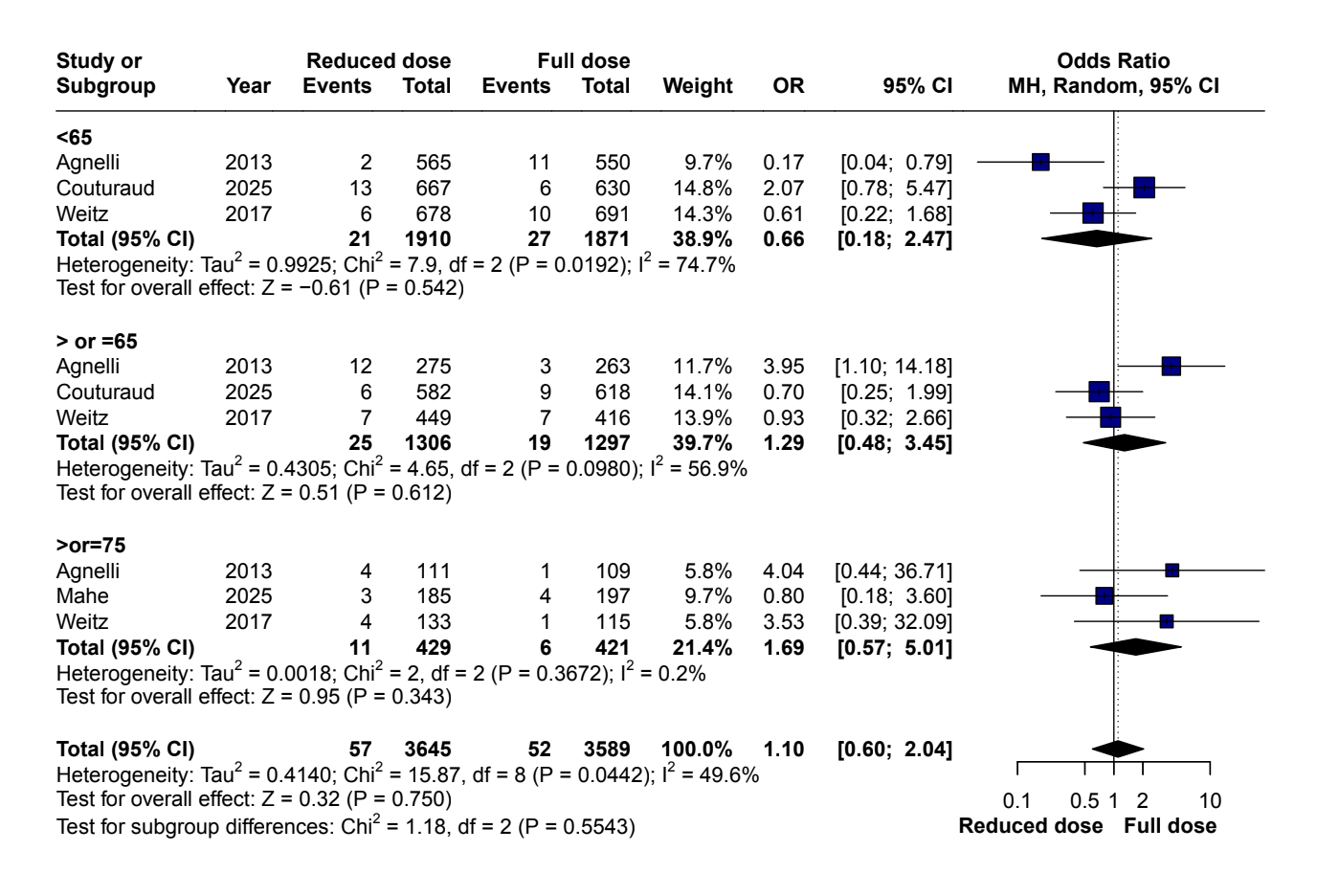
**

Figure S4: Subgroup analyses for recurrent venous thromboembolism by age. P-interaction = 0.544, indicating no significant effect modification by sex. **Ratio of ORs (<65 vs ≥65): 0.51 (95% CI 0.10–2.63)**. Absolute risk difference was -0.34% (−1.06% to 0.37%) in <65 years subgroup, +0.44% (-0.55% to 1.43%) in >=65 years subgroup and −1.13% (−0.75% to 3.01%) in >=75 years subgroup.

**Supplementary Figure S5: Subgroup analyses for recurrent Venous Thromboembolism by Body Mass Index (<30 and >=30 kg/m^2^)**


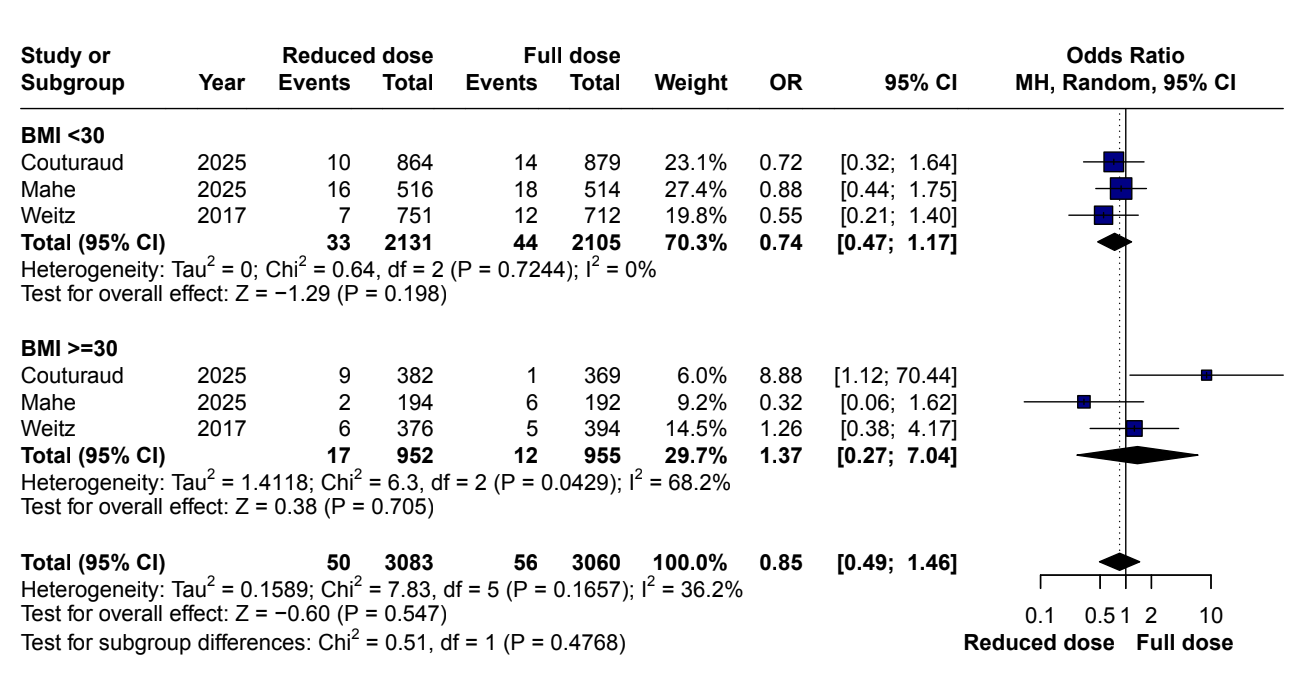


Figure S5: Subgroup analysis for recurrent venous thromboembolism by body mass index. P-interaction = 0.48, indicating no significant effect modification. Ratio of odds ratios (<30 vs ≥30 kg/m²) = 0.54 (95% CI 0.10–2.94). Absolute risk difference: −0.54% (95% CI −1.35% to +0.26%) in patients with BMI <30 kg/m²; +0.53% (95% CI −0.57% to +1.63%) in patients with BMI ≥30 kg/m².

**Supplementary Figure S6: Subgroup analyses for recurrent Venous Thromboembolism by Creatinine Clearance (<50, 50–79, and ≥80 mL/min)**


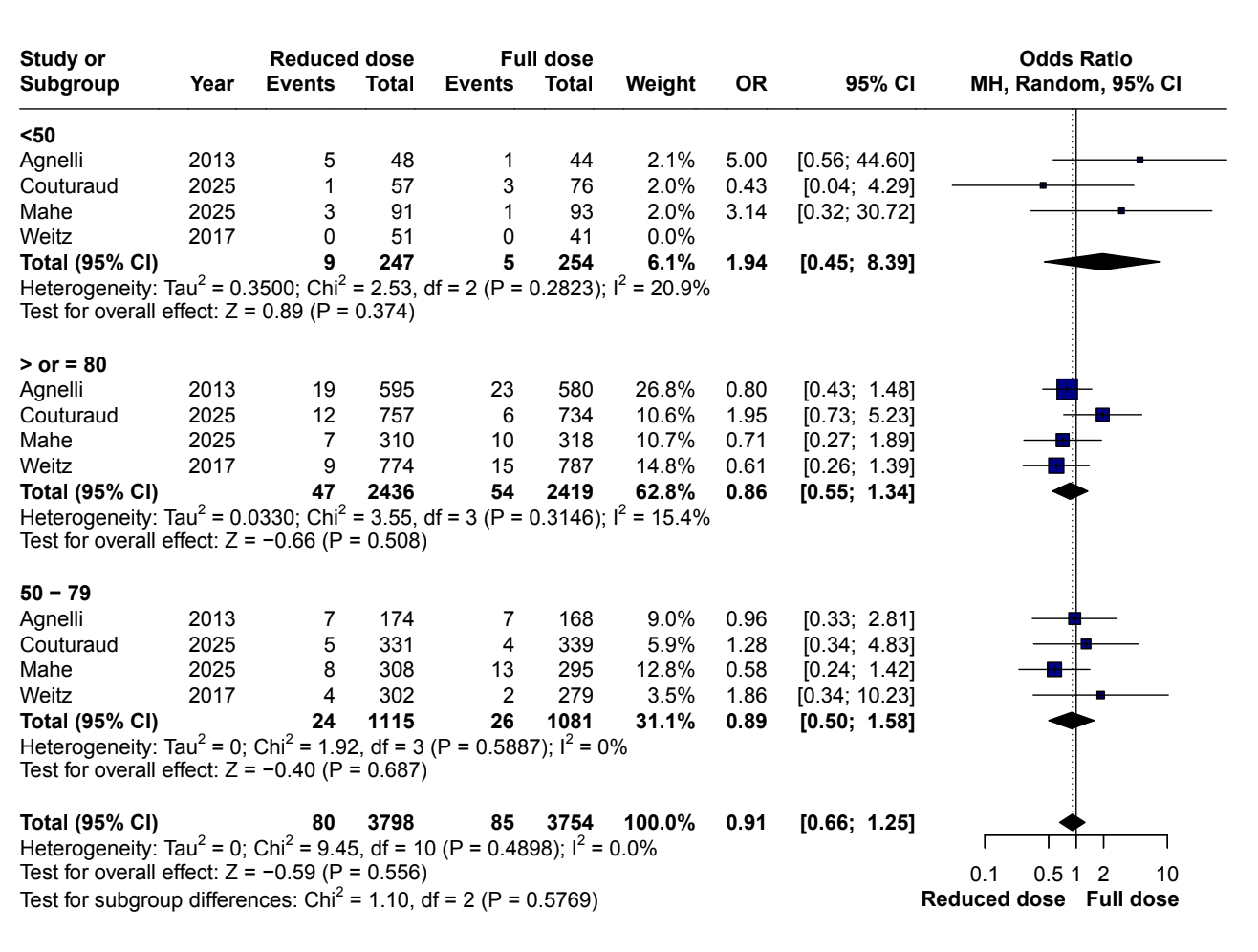


Figure S6: Subgroup analysis for recurrent venous thromboembolism by creatinine clearance. P-interaction = 0.58, indicating no significant effect modification. Absolute risk difference: +1.68% (95% CI −1.22% to +4.57%) in patients with CrCl <50 mL/min; −0.25% (95% CI −1.50% to +1.00%) in patients with CrCl 50–79 mL/min; −0.30% (95% CI −1.11% to +0.50%) in patients with CrCl ≥80 mL/min.

**Supplementary Figure S7: Subgroup analyses for recurrent Venous Thromboembolism by Active Cancer Status**


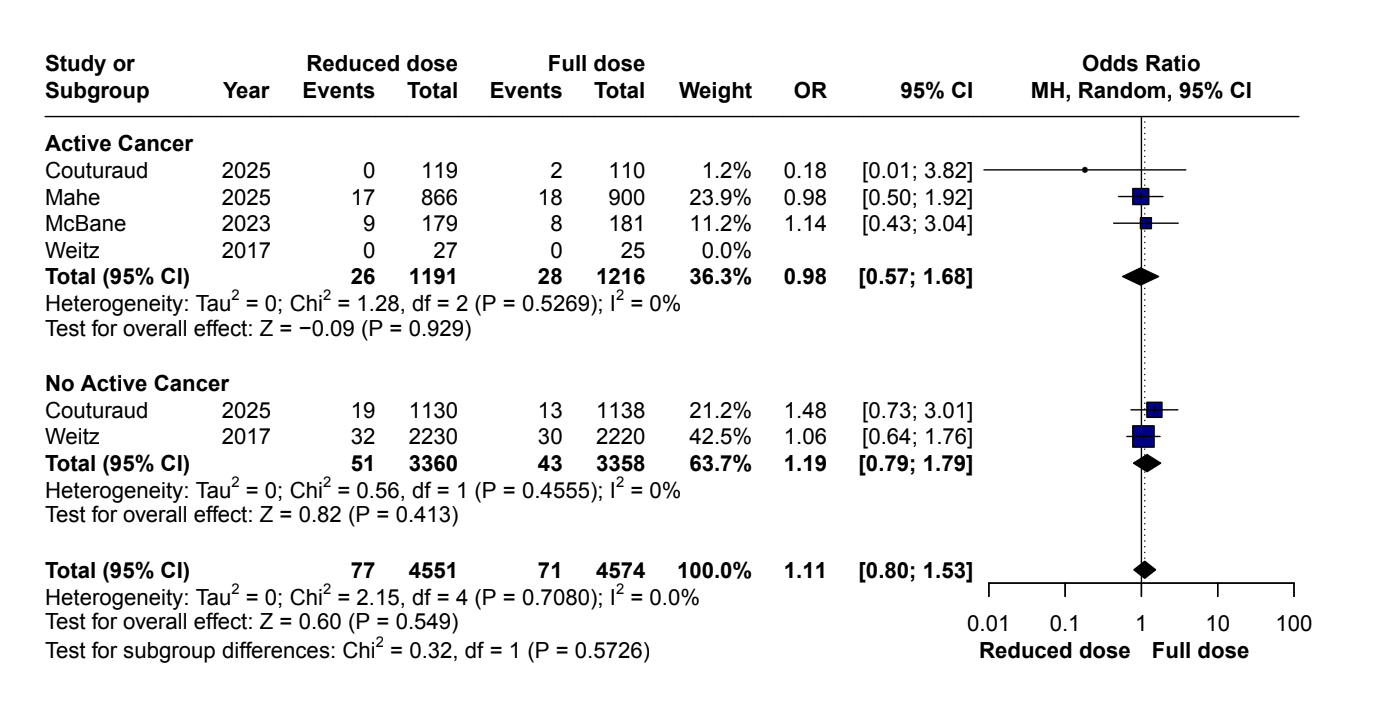


Figure S7: Subgroup analysis for recurrent venous thromboembolism by active cancer status. P-interaction = 0.57, indicating no significant effect modification. Ratio of odds ratios (active cancer vs no active cancer) = 0.82 (95% CI 0.42–1.62). Absolute risk difference: −0.12% (95% CI −1.30% to +1.06%) in patients with active cancer; +0.24% (95% CI −0.32% to +0.80%) in patients without active cancer.

**Supplementary Figure S8: Subgroup analyses for recurrent Venous Thromboembolism by Anticoagulation drug type**


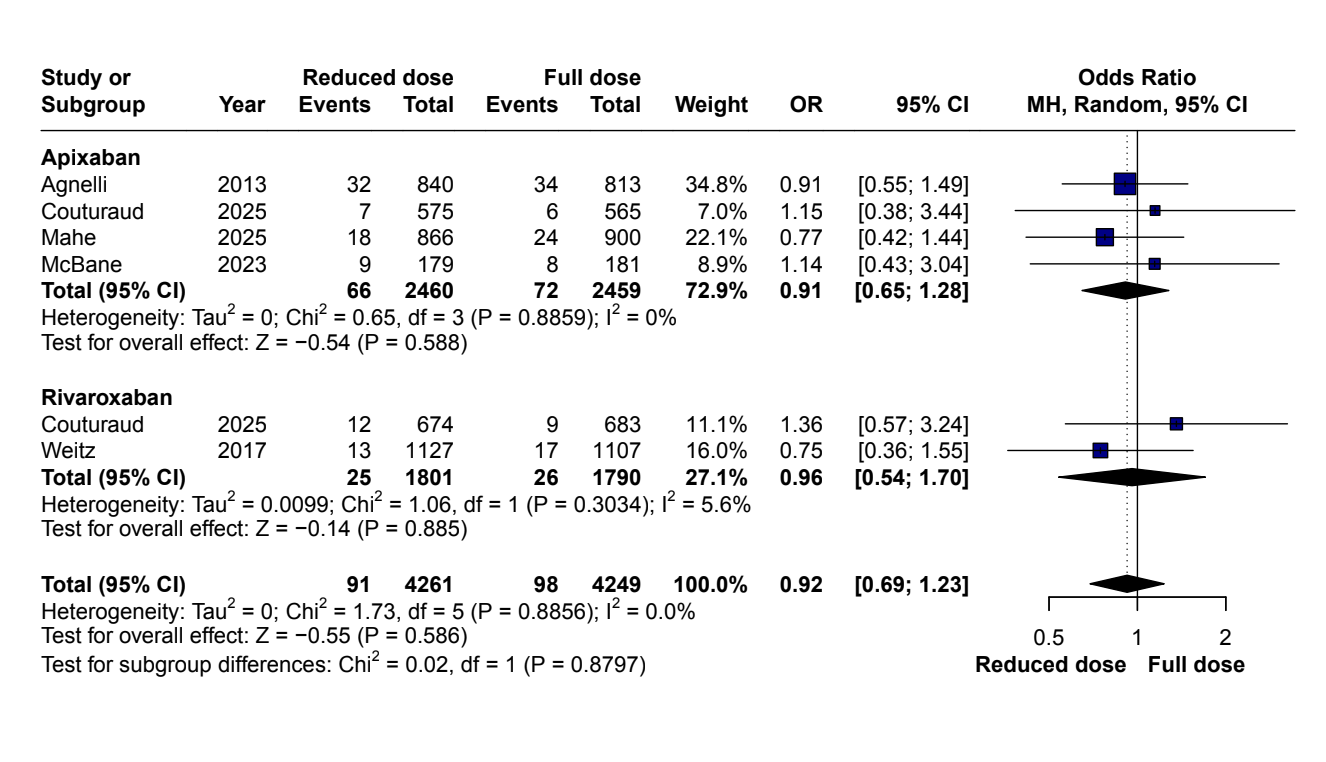


Figure S8: Subgroup analysis for recurrent venous thromboembolism by anticoagulant type. P-interaction = 0.88, indicating no significant effect modification. Ratio of odds ratios (apixaban vs rivaroxaban) = 0.95 (95% CI 0.49–1.85). Absolute risk difference: −0.25% (95% CI −1.17% to +0.68%) with apixaban; −0.06% (95% CI −0.84% to +0.71%) with rivaroxaban.

**Supplementary Figure S9: Subgroup analyses for recurrent Venous Thromboembolism by DVT status**

**
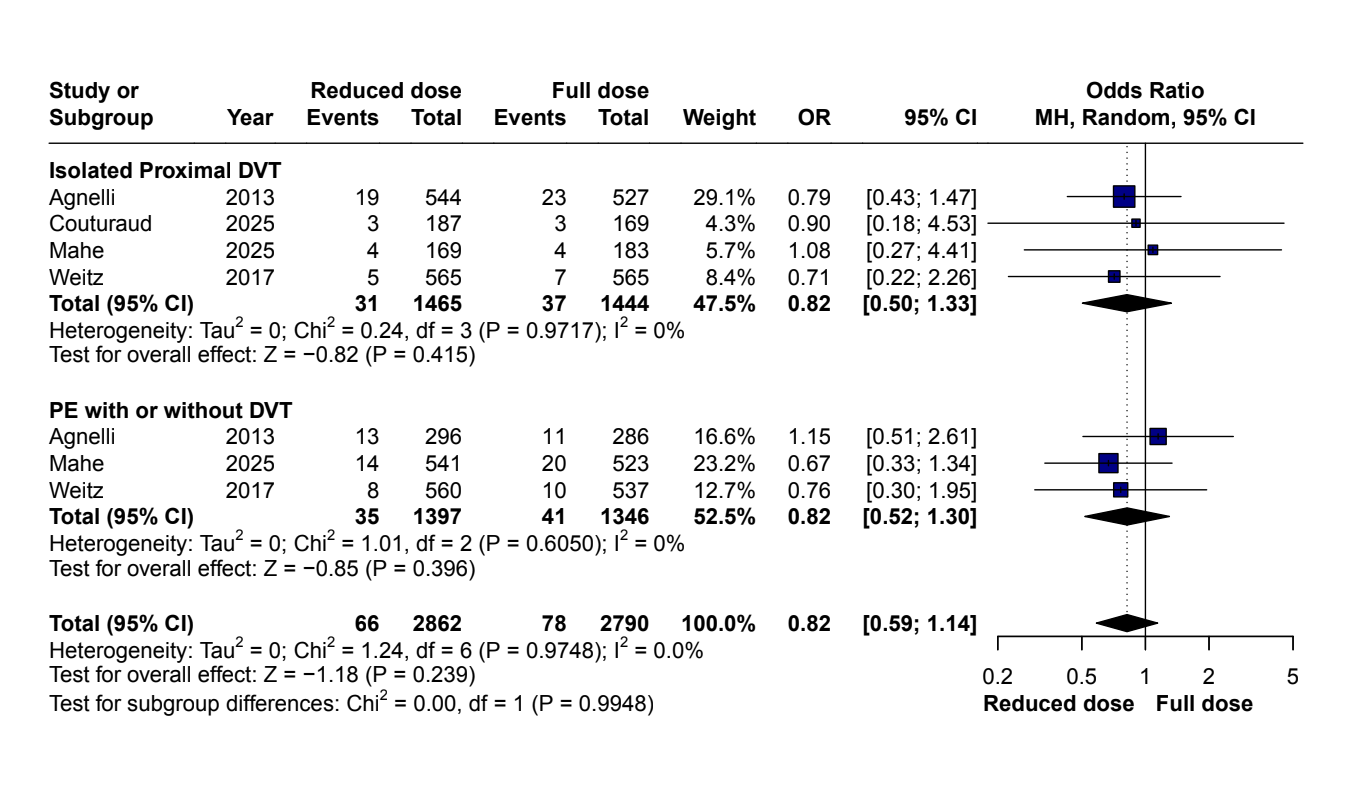
**

Figure S9: Subgroup analysis for recurrent venous thromboembolism by index event. P-interaction = 0.99, indicating no significant effect modification. Ratio of odds ratios (isolated proximal DVT vs PE with or without DVT) = 1.00 (95% CI 0.51–1.95). Absolute risk difference: −0.45% (95% CI −1.55% to +0.65%) in patients with isolated proximal DVT; −0.54% (95% CI −1.77% to +0.69%) in patients with PE with or without DVT.

**Supplementary Figure S10: Subgroup analyses for the composite of Major Bleeding and Clinically relevant Nonmajor Bleeding by Age**


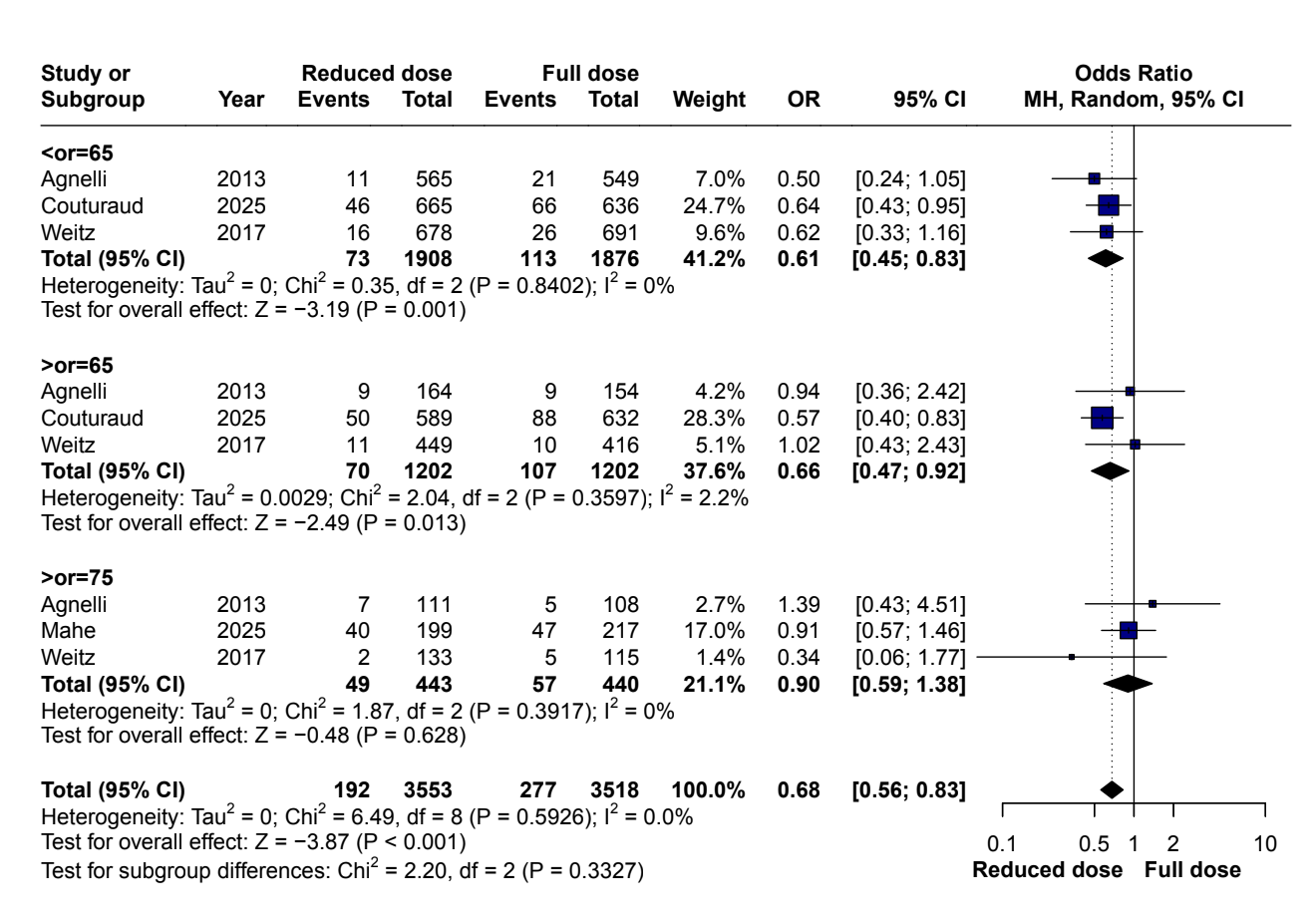


Figure S10: Subgroup analysis for the composite of major bleeding and clinically relevant non-major bleeding by age. P-interaction = 0.33, indicating no significant effect modification. Absolute risk difference: −2.20% (95% CI −3.58% to −0.82%) in patients <65 years; −3.08% (95% CI −5.16% to −0.99%) in patients ≥65 years; −1.89% (95% CI −6.18% to +2.39%) in patients ≥75 years.

**Supplementary Figure S11: Subgroup analyses for the composite of Major Bleeding and Clinically relevant Nonmajor Bleeding by Sex**


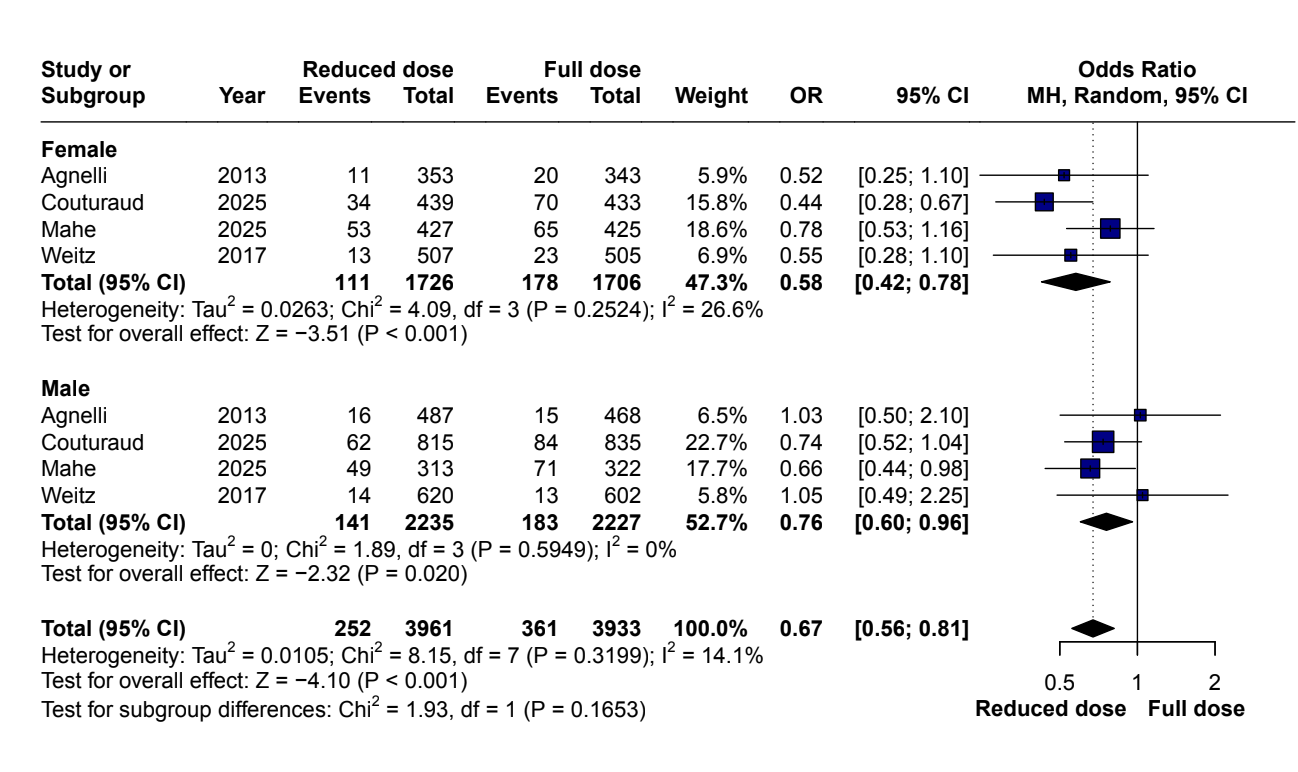


Figure S11: Subgroup analysis for the composite of major bleeding and clinically relevant non-major bleeding by sex. P-interaction = 0.165, indicating no significant effect modification. Ratio of odds ratios (female vs male) = 0.76 (95% CI 0.52–1.13). Absolute risk difference: −4.00% (95% CI −5.86% to −2.15%) in females; −1.91% (95% CI −3.43% to −0.39%) in males.

**Supplementary Figure S12: Subgroup analyses for the composite of Major Bleeding and Clinically relevant Nonmajor Bleeding by Creatinine Clearance (<50, 50–79, and ≥80 mL/min)**


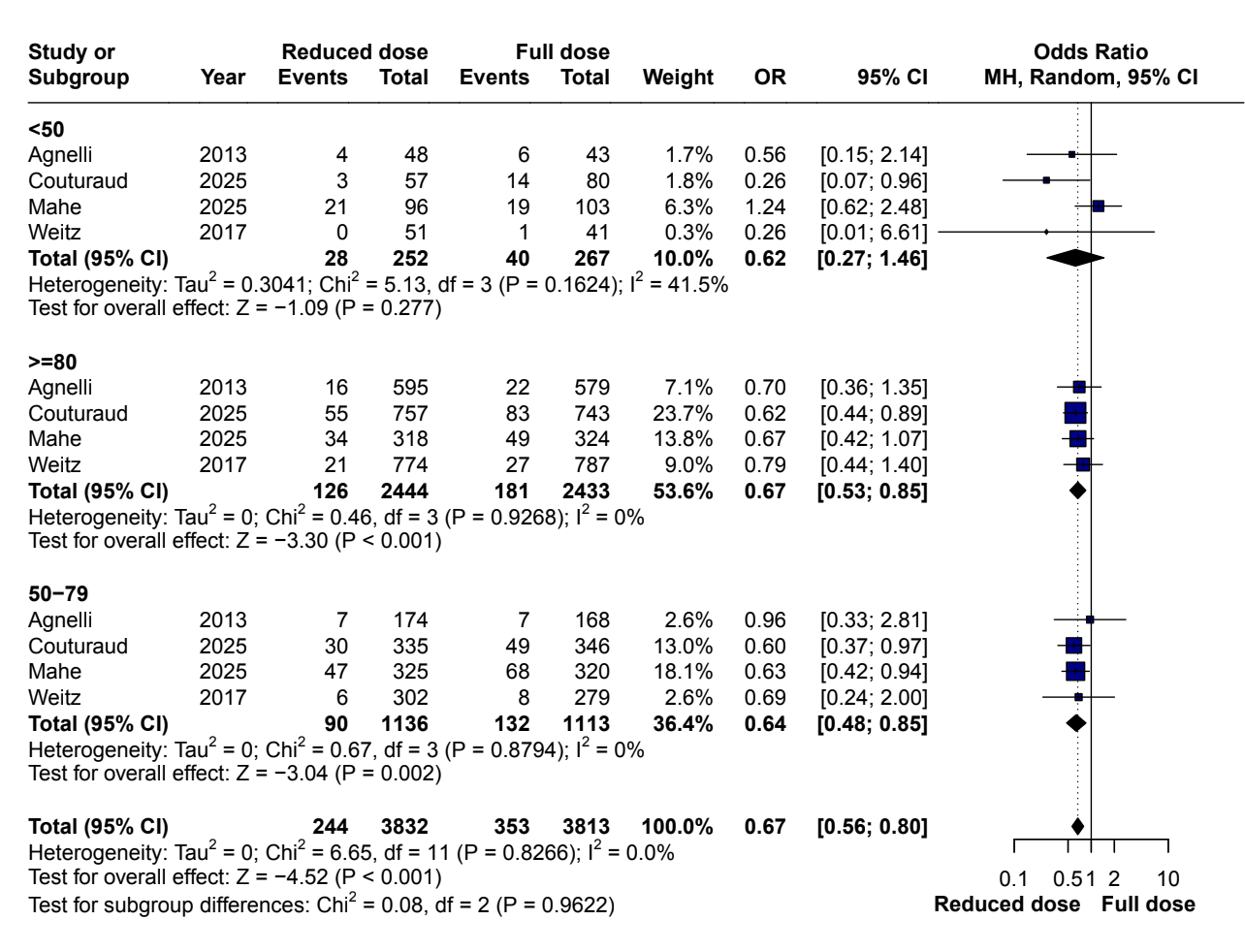


Figure S12: Subgroup analysis for the composite of major bleeding and clinically relevant non-major bleeding by creatinine clearance. P-interaction = 0.96, indicating no significant effect modification. Absolute risk difference: −3.87% (95% CI −9.65% to +1.91%) in patients with CrCl <50 mL/min; −3.94% (95% CI −6.40% to −1.47%) in patients with CrCl 50–79 mL/min; −2.28% (95% CI −3.65% to −0.92%) in patients with CrCl ≥80 mL/min.

**Supplementary Figure S13: Subgroup analyses for the composite of Major Bleeding and Clinically relevant Nonmajor Bleeding by Active Cancer**


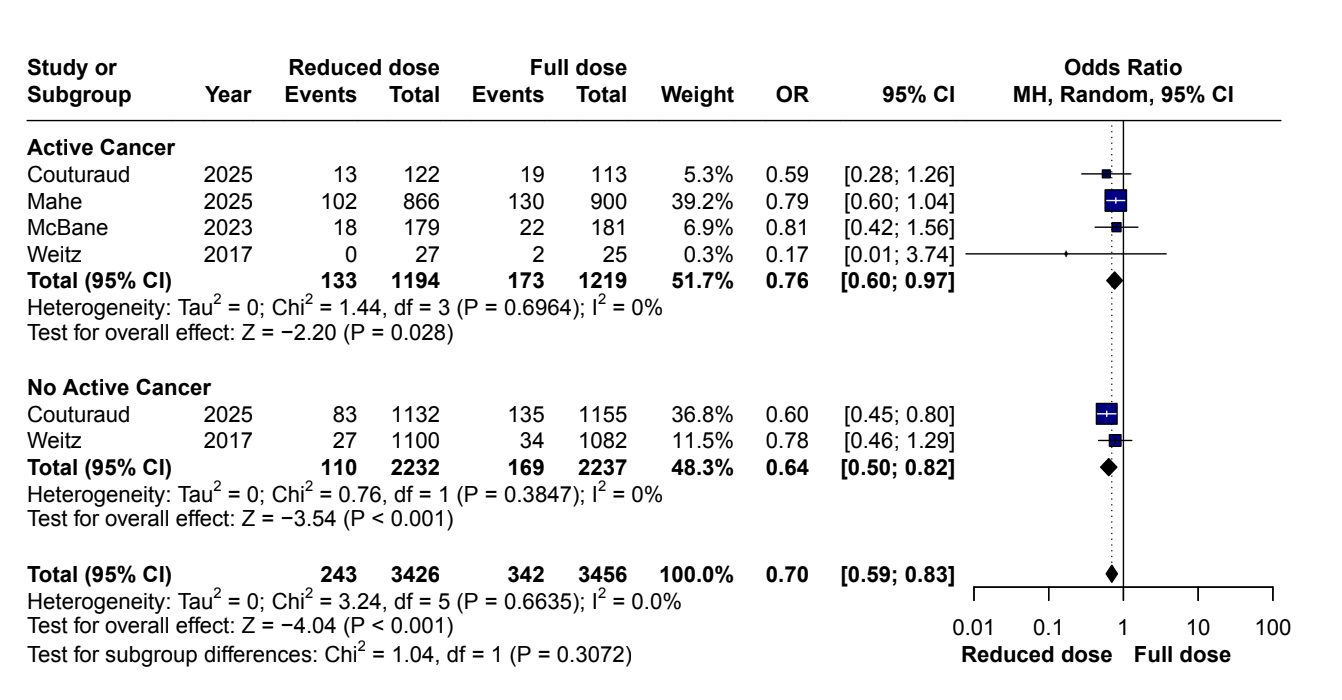


Figure S13: Subgroup analysis for the composite of major bleeding and clinically relevant non-major bleeding by active cancer status. P-interaction = 0.31, indicating no significant effect modification. Ratio of odds ratios (active cancer vs no active cancer) = 1.19 (95% CI 0.84–1.68). Absolute risk difference: −3.05% (95% CI −5.70% to −0.40%) in patients with active cancer; −2.63% (95% CI −4.04% to −1.21%) in patients without active cancer.

**Supplementary Figure S14: Subgroup analyses for the composite of Major Bleeding and Clinically relevant Nonmajor Bleeding by Anticoagulation Drug Type**


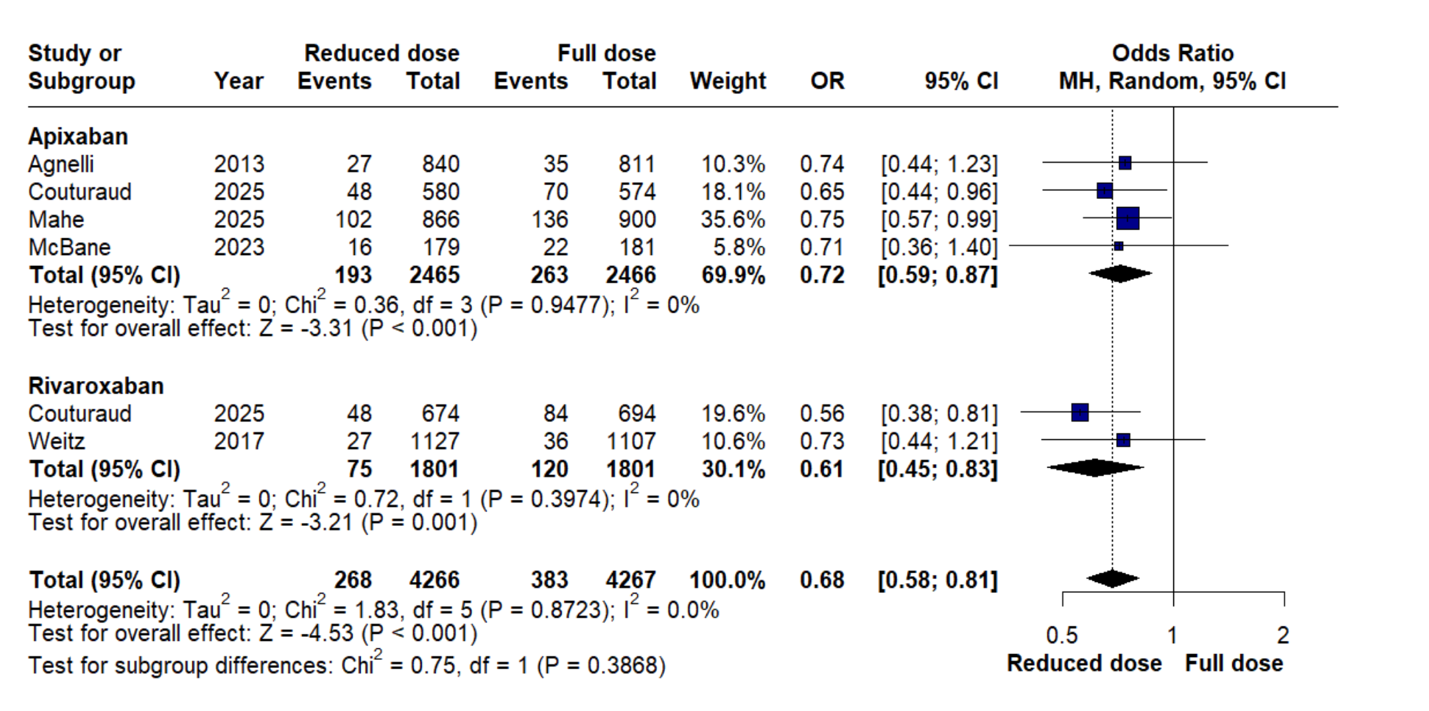


Figure S14: Subgroup analysis for the composite of major bleeding and clinically relevant non-major bleeding by anticoagulant type. P-interaction = 0.39, indicating no significant effect modification. Ratio of odds ratios (apixaban vs rivaroxaban) = 1.18 (95% CI 0.82–1.70). Absolute risk difference: −2.84% (95% CI −4.45% to −1.22%) with apixaban; −2.50% (95% CI −3.97% to −1.02%) with rivaroxaban.

**Supplementary Figure S15: Subgroup analyses for the composite of Major Bleeding and Clinically relevant Nonmajor Bleeding by DVT status**


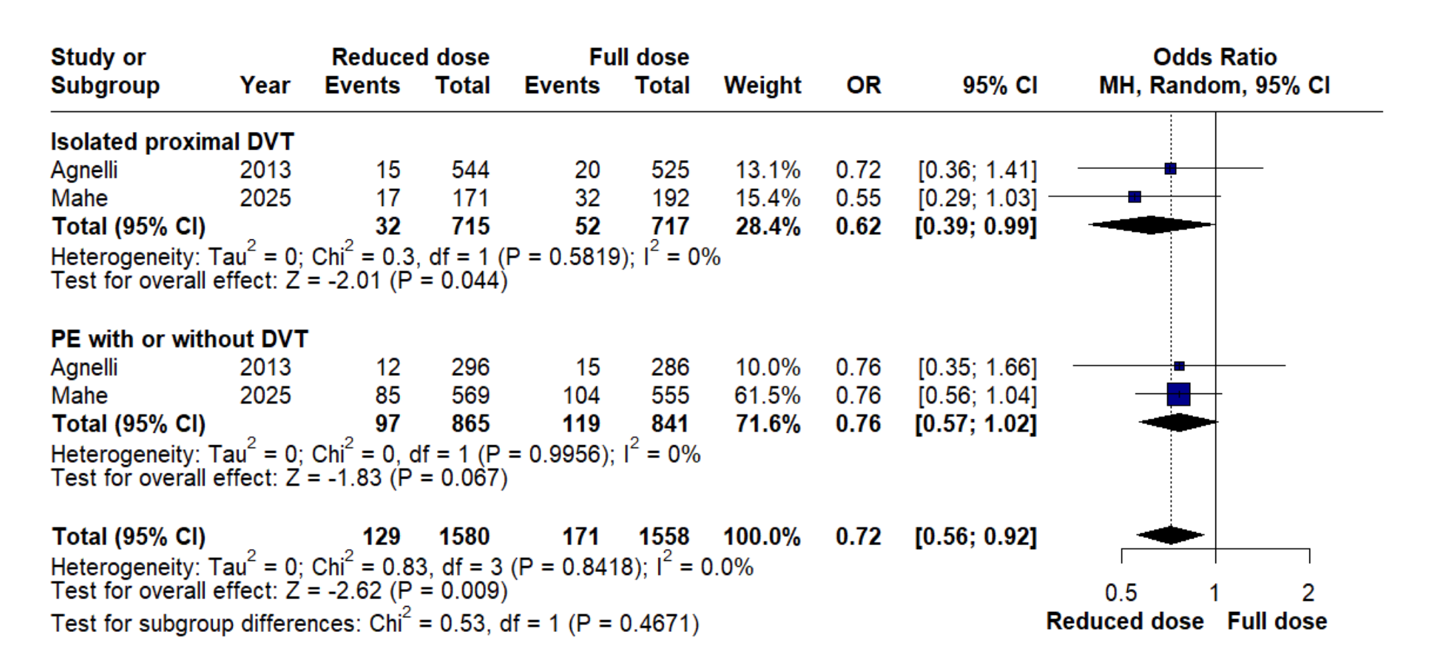


Figure S15: Subgroup analysis for the composite of major bleeding and clinically relevant non-major bleeding by index event. P-interaction = 0.47, indicating no significant effect modification. Ratio of odds ratios (isolated proximal DVT vs PE with or without DVT) = 0.82 (95% CI 0.47–1.41). Absolute risk difference: −2.78% (95% CI −5.21% to −0.35%) in patients with isolated proximal DVT; −2.94% (95% CI −6.09% to +0.22%) in patients with PE with or without DVT.

**Supplementary Figure S16: Subgroup Analysis for Recurrent Venous Thromboembolism**

**
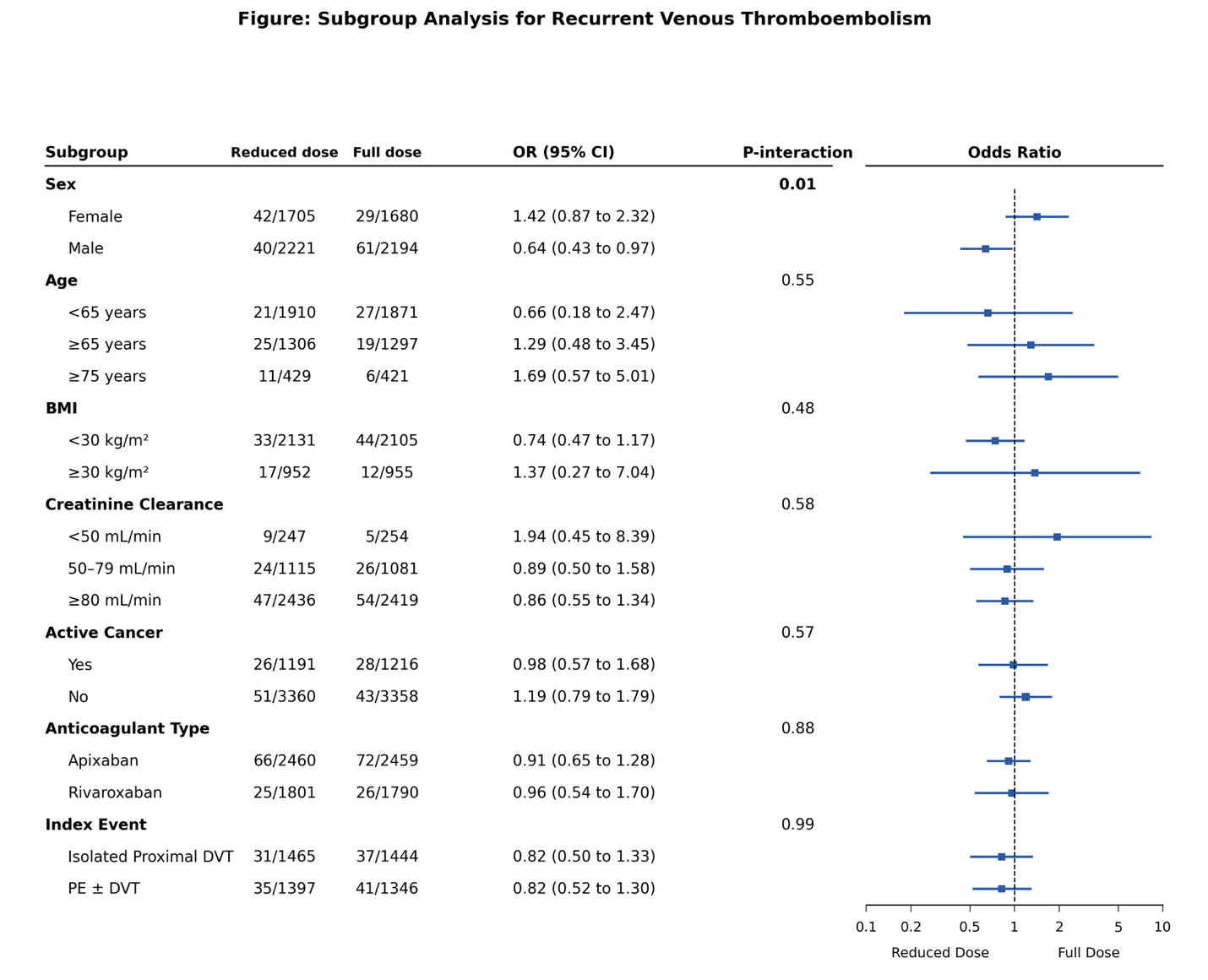
**

**Supplementary Figure S17: Subgroup Analysis for Composite Bleeding Outcome
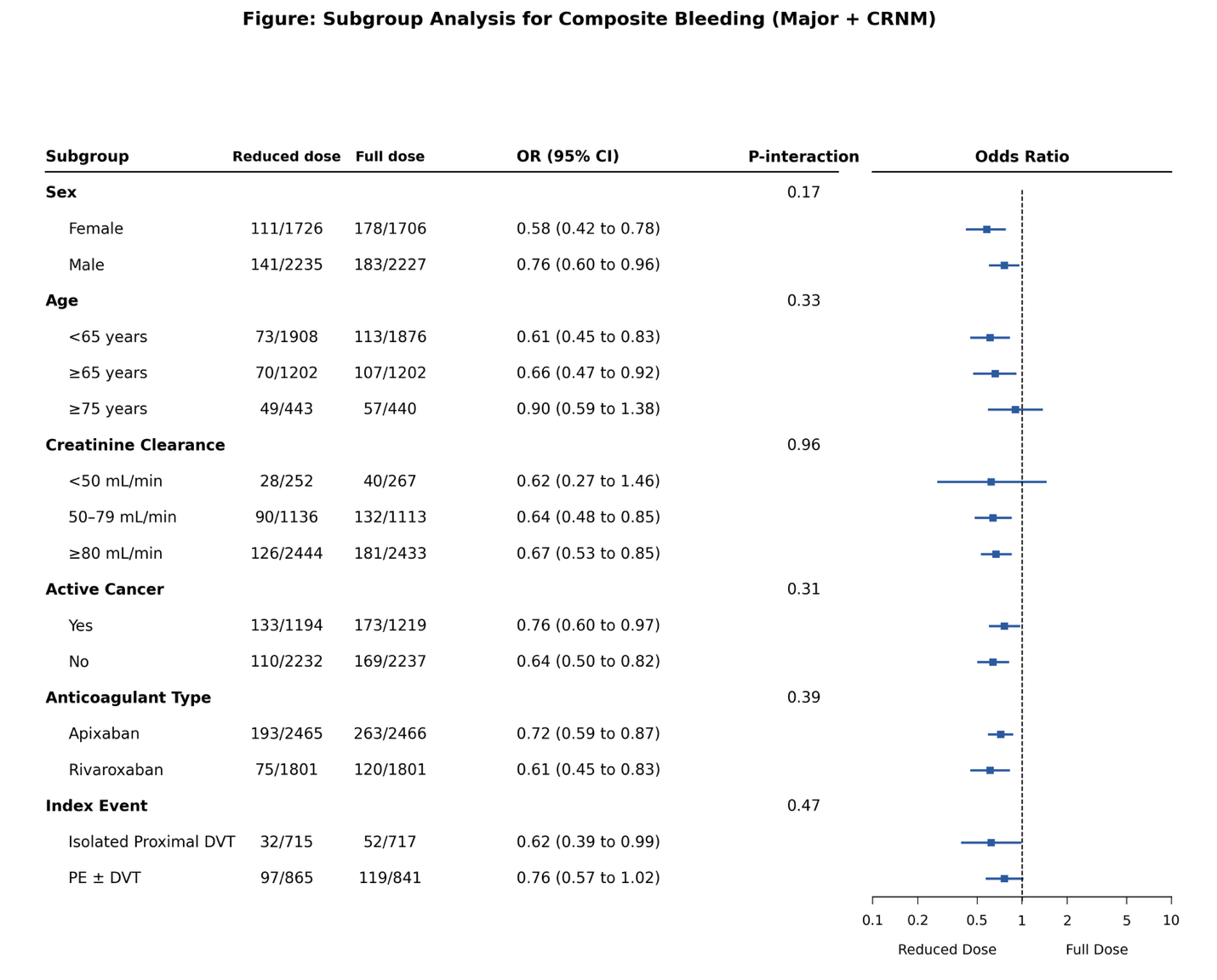
**

**Supplementary Figure S18: Leave-one-out sensitivity analyses**


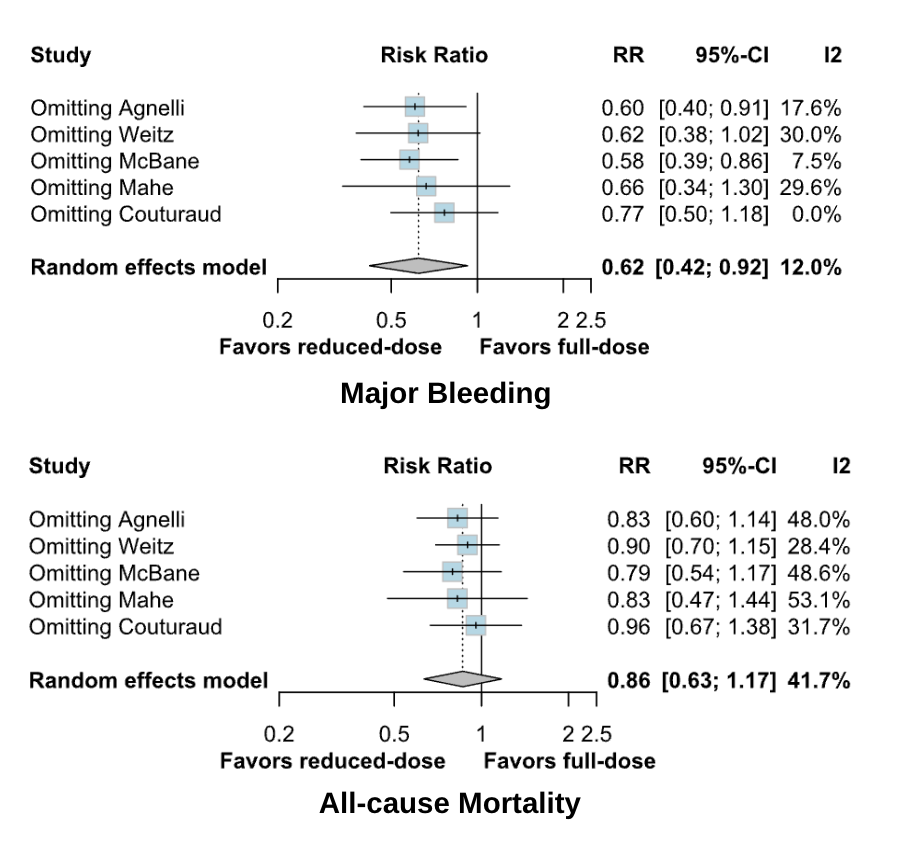


**Supplementary Figure S19: Meta-regression Analyses of recurrent VTE (RR) with follow-up duration as the covariate**


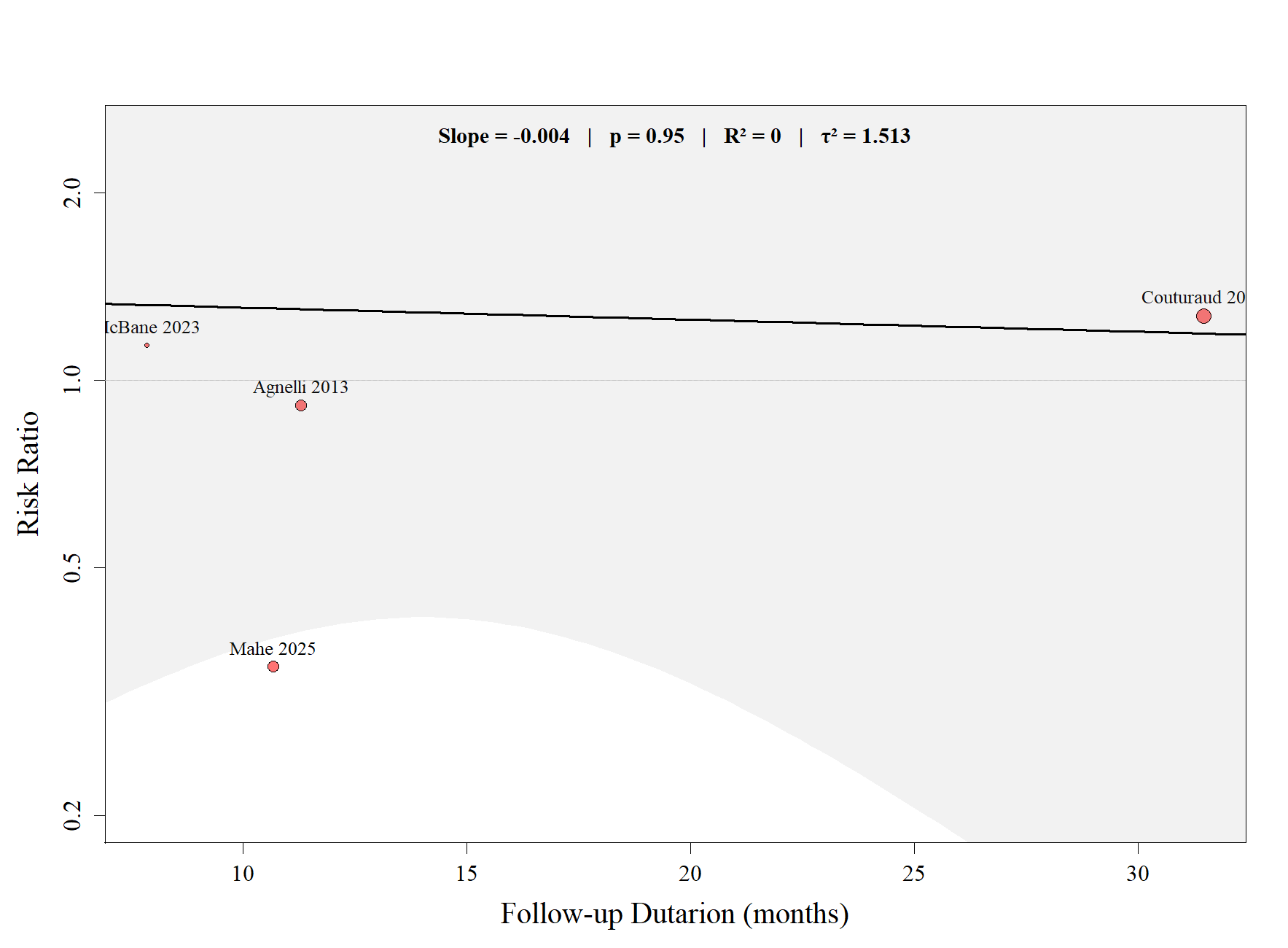


**Supplementary Figure S20: Meta-regression Analyses of major bleeding (RR) with follow-up duration as the covariate**


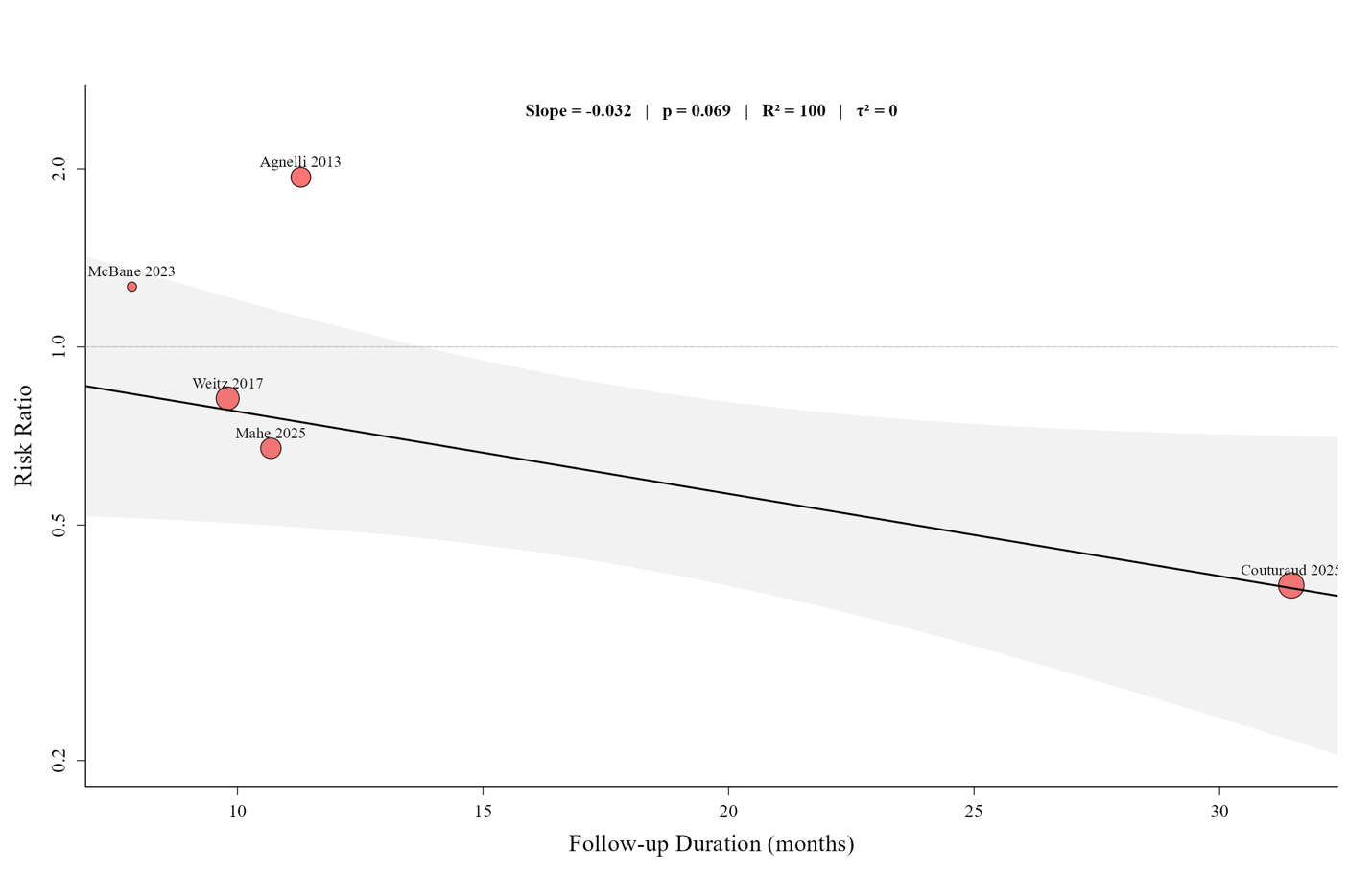


**Supplementary Figure S21: Meta-regression Analyses of Clinically relevant Nonmajor bleeding (RR) with follow-up duration as the covariate**


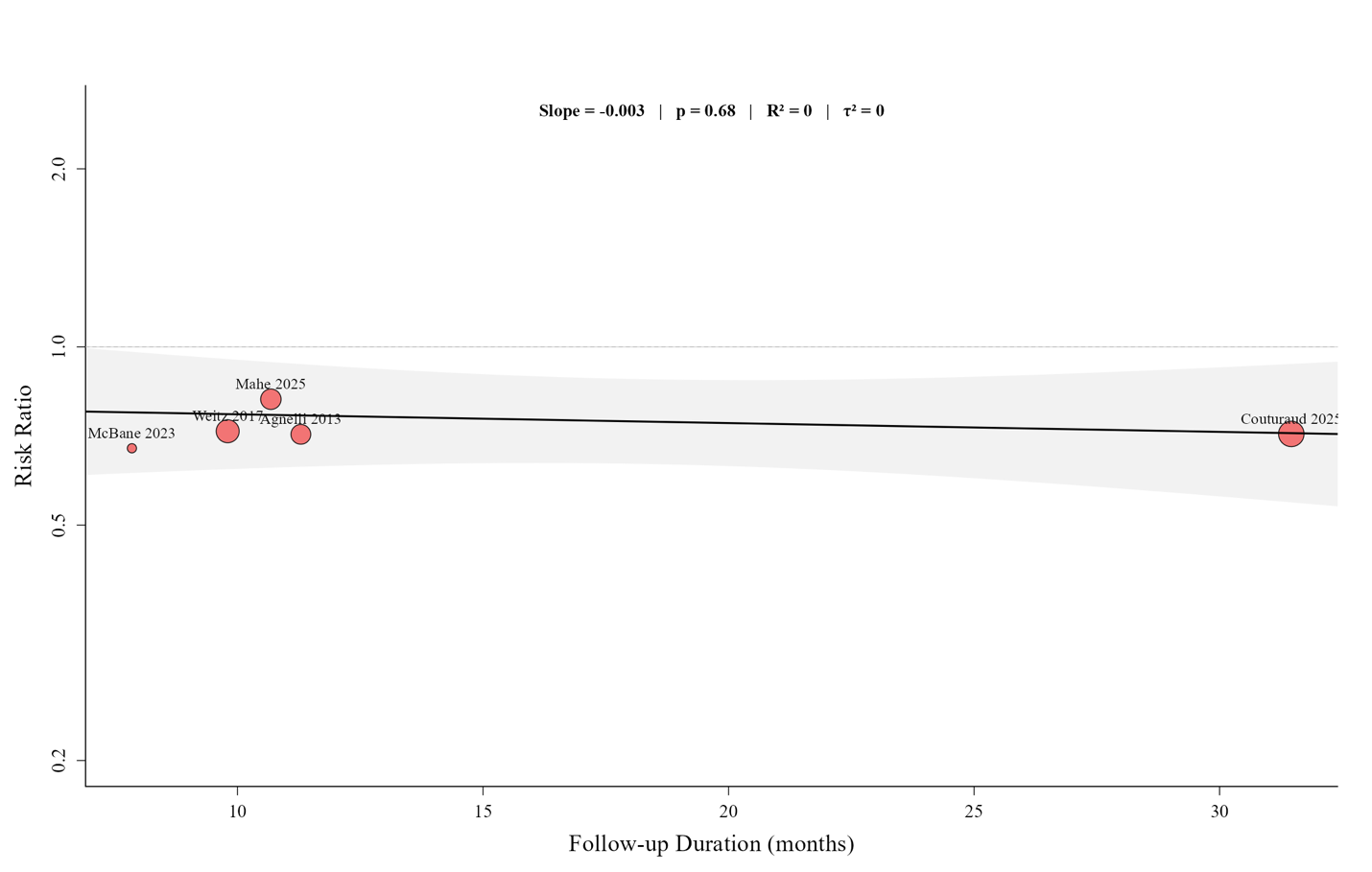


**Supplementary Figure S22: Meta-regression Analyses of Fatal Bleeding (RR) with follow-up duration as the covariate**


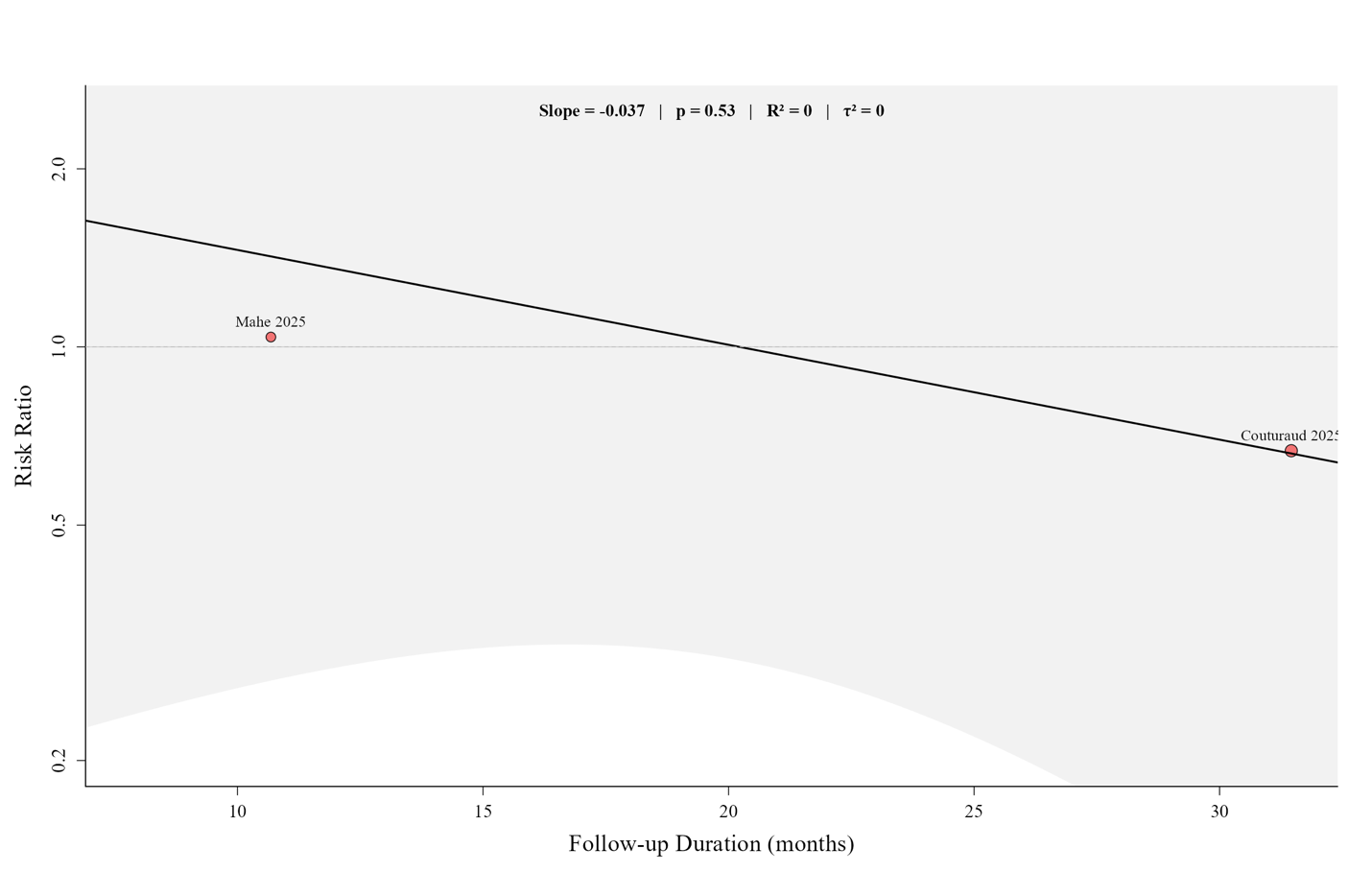


**Supplementary Figure S23: Meta-regression Analyses of Major Bleeding (HR) with follow-up duration as the covariate**


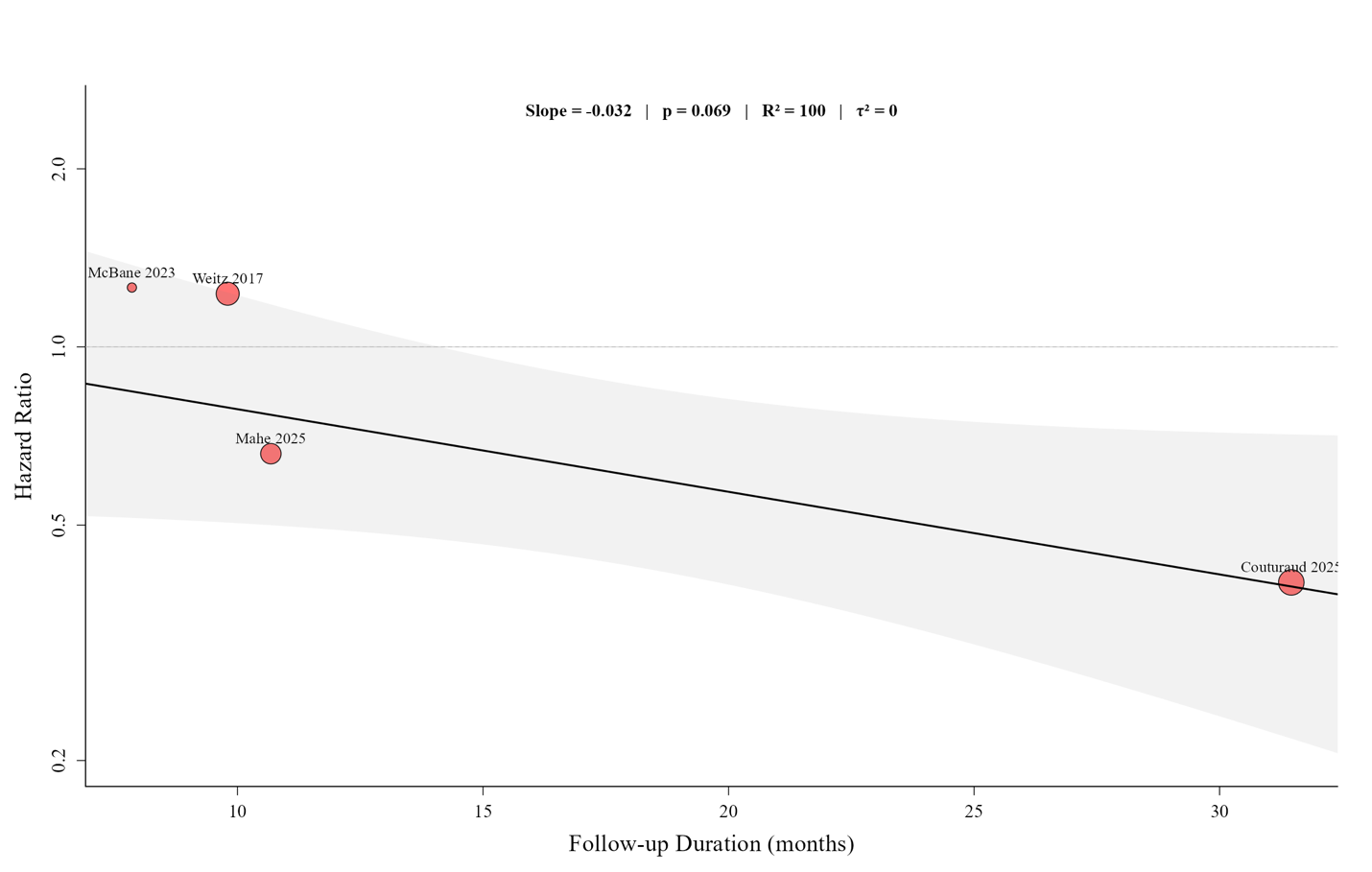


**Supplementary Figure S24: Meta-regression Analyses of Composite of Major Bleeding and Clinically relevant Nonmajor bleeding (RR) with follow-up duration as the covariate**


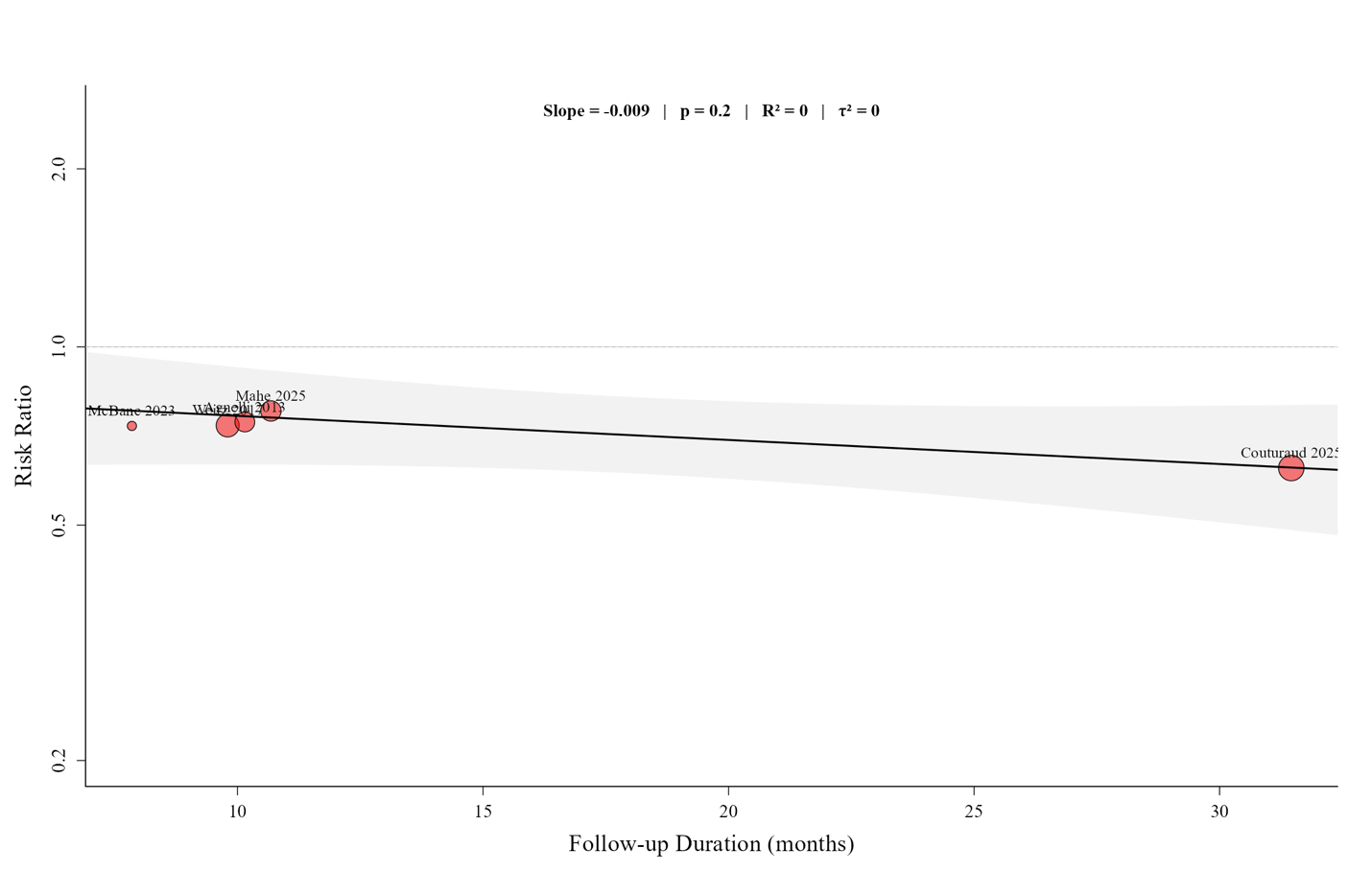


**Supplementary Figure S25: Meta-regression Analyses of Mortality (RR) with follow-up duration as the covariate**

**
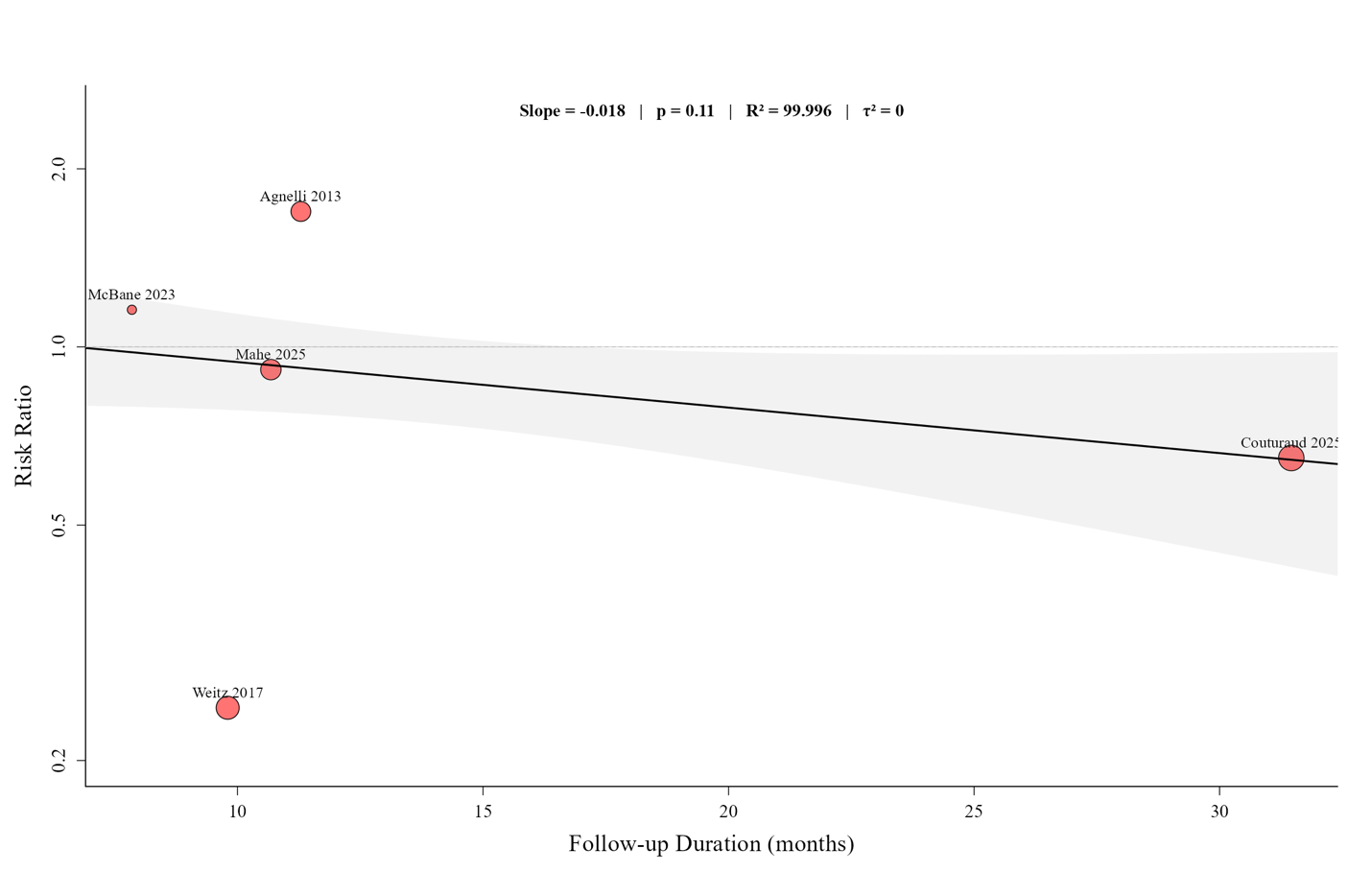
**

**Supplementary Figure S26: Meta-regression Analyses of Pulmonary Embolism (RR) with follow-up duration as the covariate**

**
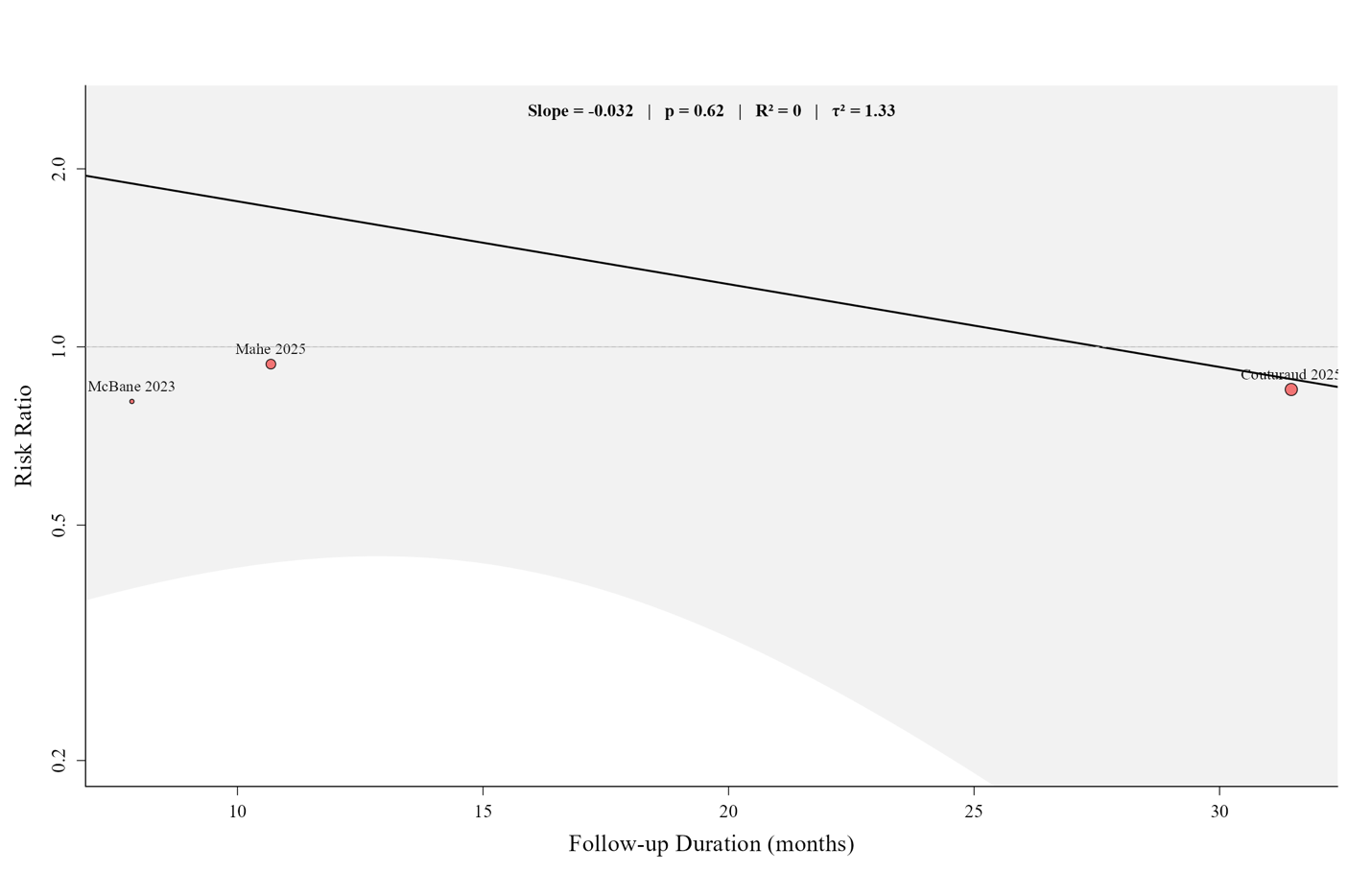
**
